# Multi-omics analysis reveals the association between elevated KIF18B expression and unfavorable prognosis, immune evasion, and regulatory T cell activation in nasopharyngeal carcinoma

**DOI:** 10.3389/fimmu.2023.1258344

**Published:** 2023-09-08

**Authors:** Siqi Tang, Zhenyu Wu, Lusi Chen, Longjiang She, Weihan Zuo, Weijun Luo, Yang Zhang, Shaoqiang Liang, Guichao Liu, Biyi He, Jinfeng He, Ning Zhang

**Affiliations:** ^1^Department of Radiation Oncology, First People’s Hospital of Foshan, Foshan, China; ^2^Department of Urology, First People’s Hospital of Foshan, Foshan, China; ^3^Department of Comprehensive (Head and Neck) Oncology and Hospice Ward, First People’s Hospital of Foshan, Foshan, China; ^4^Department of Rhinology, First People’s Hospital of Foshan, Foshan, China

**Keywords:** KIF18B, nasopharyngeal carcinoma, regulatory T cell, immune infiltration, intercellular communication, epithelial-mesenchymal transition, m6A modification

## Abstract

**Background:**

Nasopharyngeal carcinoma (NPC) is prevalent in Southern China. The expression profile and functions of kinesin family member 18B (KIF18B) remain unclear in NPC.

**Methods:**

Bulk and single-cell transcriptome data for NPC were downloaded. KIF18B expression differences in NPC and normal tissues and its prognostic value were validated by immunohistochemistry and Cox model. We performed multi-faceted functional enrichment analysis on KIF18B. Immune infiltration was analyzed comprehensively by the CIBERSORT, EPIC, and quanTIseq algorithms and the BisqueRNA package and confirmed by immunofluorescence assay. The intercellular communication were investigated by the CellChat package. We explored the dynamics of KIF18B expression by pseudotime trajectory. M6A modification analysis rely on SRAMP platform. The treatment response were evaluated by Tumor Immune Dysfunction and Exclusion (TIDE) score, immunophenoscore and IC50 value.

**Results:**

KIF18B overexpression in NPC led to unfavorable prognosis, and significantly associated with advanced T, N, and stage classifications. Functional analysis demonstrated that KIF18B was involved in immune suppression, epithelial-mesenchymal transition (EMT), N6-methyladenosine (m6A) modification and therapeutic responses. The deconvolution algorithm indicated that activated regulatory T cells (Tregs) had the strongest positive correlation with KIF18B among immune cells (R = 0.631). Validated by immunofluorescence assay, the high KIF18B expression group displayed a notable rise in Tregs infiltration, accompanied by a substantial decrease in the infiltration of CD8^+^ T cells and macrophages. In the intercellular communication network, malignant cells with high KIF18B expression implicated in more interactions, and activated and recruited Tregs by modulating cytokines, chemokines, and immune checkpoints. KIF18B was upregulated in more advanced malignant cells and influenced EMT by regulating ITGA6, VIM, and ZEB1/2. KIF18B expression was positively related to m6A “writer” and “reader” genes, and negatively related to “eraser” genes. The KIF18B high expression group exhibited a higher TIDE score and elevated IC50 values for the commonly used chemotherapy drugs, gemcitabine, oxaliplatin, and 5-fluorouracil.

**Conclusion:**

KIF18B is a significant prognostic marker in NPC, and may modulate immune evasion and EMT. M6A modification may account for the aberrant overexpression of KIF18B in NPC. Furthermore, KIF18B may predict response to immunotherapy and chemotherapy.

## 1 Introduction

Nasopharyngeal carcinoma (NPC), which has a distinct geographical distribution, is endemic to Southern China and Southeast Asia. In 2018, approximately 130,000 new cases of NPC were reported worldwide, of which China accounted for around 50%, with an age-standardized incidence rate of 3/100,000 (1). Application of intensity-modulated radiotherapy and optimized chemotherapy has led to an improvement in the survival rate of patients with NPC. However, the median progression-free survival (PFS) for recurrent or metastatic disease after first-line platinum-based treatment is only about 7 months (2). Of the existing therapeutics, immune checkpoint therapy appears to have achieved a breakthrough (1) with an objective response rate of 20-34% (3–6). Nevertheless, a large proportion of patients do not benefit from immunotherapy, due to the development of immune escape by malignant cells. Thus, it is crucial to explore the potential metastatic mechanisms that are involved as well as the status of the NPC tumor microenvironment (TME).

Kinesin family member 18B (KIF18B) is a microtubule motor protein belonging to the kinesin-8 superfamily that plays an important role in chromosomal separation and positioning during cell division (7). Evidently, KIF18B contributes to the malignant behavior of tumors. For instance, KIF18B overexpression in sarcomas led to decreased radiosensitivity (8). In prostate cancer, KIF18B enhanced cell proliferation, migration, and invasion, and suppressed apoptosis (9). KIF18B dysregulation was also found to be associated with immune evasion-related factors, such as microsatellite instability and mismatch repair (10). Thus, KIF18B may act as a key oncogene and potential therapeutic target in cancers. However, the role of KIF18B in NPC remains unclear.

**Figure 1:**
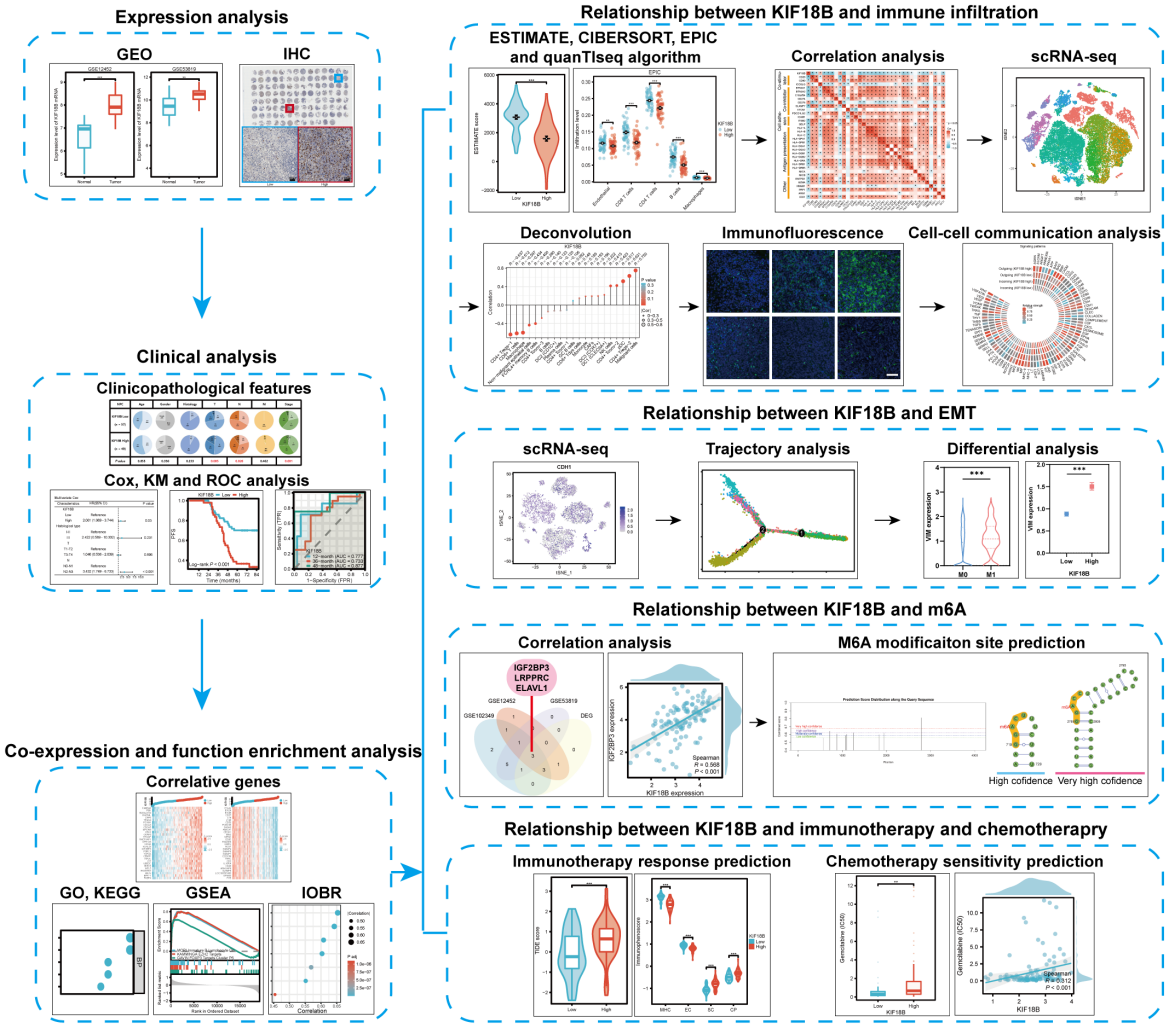
Schematic diagram of the study design.

Using bulk RNA sequencing (RNA-seq) and single-cell RNA-seq (scRNA-seq) data, as well as basic experiments, we investigated the expression of KIF18B in NPC and assessed its association with clinical outcomes, tumorigenic mechanisms, immune escape, epithelial-mesenchymal transition (EMT), N6-methyladenosine (m6A) modification, and therapeutic responses. We also explored the influence of KIF18B on intercellular communication within the TME. The study design is illustrated (**Figure 1**).

## 2 Method

### 2.1 Data collection and processing

The transcriptomic and clinical data of three datasets, GSE12452, GSE53819, and GSE102349, were retrieved from the gene expression omnibus (GEO) database (https://www.ncbi.nlm.nih.gov/geo/; **Supplementary Tables S1****-** **3**). R software was utilized to perform data integration, analysis, and visualization. The VLOOKUP function in Excel was applied to align and combine data. We used the limma package to detect differentially expressed genes (DEGs) between non-malignant nasopharyngeal and NPC tissues (11). Based on the median expression of KIF18B, the NPC samples from the GSE102349 cohort were stratified into two groups: high KIF18B expression; and low KIF18B expression.

### 2.2 Clinical sample collection

Formalin-fixed, paraffin-embedded clinical samples comprising 29 NPC tissues and 16 adjacent or normal nasopharyngeal tissues were obtained from the specimen bank of First People’s Hospital of Foshan for differential expression analysis (**Supplementary Table S4**). None of these patients had undergone any systemic anti-tumor treatment prior to biopsy. These tissues were sliced into 3μm thick consecutive sections, and subjected to immunohistochemical (IHC) staining. A human NPC tissue microarray (Outdo Biotech, Shanghai, China) was used to examine the role of KIF18B in NPC via IHC. The microarray consisted of 110 NPC samples resected between January 2010 and October 2011 (**Supplementary Table S5**). Clinical samples were subjected to tumor, lymph node, and metastasis staging using the 8th edition of the Union for International Cancer Control/American Joint Committee on Cancer staging system. Written informed consent was obtained from all patients. This study was approved by the Ethics Committee of First People’s Hospital of Foshan.

### 2.3 Immunohistochemistry assays

After dewaxing and hydration, endogenous peroxidases in the tissue sections were blocked with a hydrogen peroxide blocking solution at room temperature for 15 min. The sections were incubated in an antigen retrieval solution (H3300; Vector Labs, Newark, CA 94560, USA), and antigen retrieval was performed in a microwave oven. Nonspecific antigens were blocked with goat serum (AR0009; Boster, Wuhan, China) for 1 h. The sections were incubated with rabbit anti-KIF18B primary antibody (1:50, ab121798, Abcam) in a humidified container at 4°C overnight. The next day, the slides were incubated with anti-rabbit HRP-labeled secondary antibody in the dark at 37°C for 20 min. The sections were stained with DAB and hematoxylin. Hydrogen peroxide blocking solution and enzyme-labeled goat anti-rabbit IgG polymer were obtained from a rabbit two-step kit (PV-6001, ZSGB-BIO, Beijing, China). Images were acquired under microscopy (Olympus, Tokyo, Japan).

Two pathologists who specialized in NPC and were unaware of any detailed information regarding the patients involved, independently evaluated IHC staining. Samples with > 50% tissue loss or tissue folding that interfered with post-IHC staining assessments were discarded, resulting in 106 tissue microarray samples being included in the final analysis. A semi-quantitative immunoreactive score was used to evaluate the expression levels of KIF18B (12). The staining intensity of KIF18B in the NPC tissues was scored as 0 (negative), 1 (weak), 2 (moderate), or 3 (strong). The percentage of stained cells was scored as follows: 0 (no staining), 1 (1-10%), 2 (11-50%), 3 (51-80%), and 4 (81-100%). The final score for each section was calculated by multiplying intensity and proportion scores. Receiver operating characteristic (ROC) analysis was used to rank the patients based on the cut-off value of KIF18B expression for PFS outcome; thus, all clinical samples in the tissue microarray were classified into two groups (low KIF18B expression and high KIF18B expression groups).

### 2.4 Functional enrichment analysis

The limma R package was used to identify DEGs between the KIF18B high- and low-expression groups in the GSE102349 dataset (11). We used the criteria |logFC| > 1, adjust *P*-value < 0.05, and |R| > 0.7 to select a subset of DEGs that showed strong correlation with KIF18B expression. The genes of this subset underwent Gene Ontology (GO) and Kyoto Encyclopedia of Genes and Genomes (KEGG) pathway analysis using the clusterProfiler R package (13). To further explore the potential mechanism underlying the role of KIF18B, we conducted gene set enrichment analysis (GSEA) through the clusterProfiler R package (13) and reference gene sets from the Molecular Signatures Database (MSigDB) (14). The hallmark and curated gene collections were used as annotated gene sets. The IOBR package is a newly developed software package that contains 255 novel feature gene sets mainly published in 2018 covering tumor metabolism, m6A, and tertiary lymphoid structures (15). Therefore, we explored the relationship between KIF18B and novel phenotypes by applying the signature estimation function in the R package using single-sample GSEA (ssGSEA).

### 2.5 Immune infiltration analysis based on bulk RNA-seq data

Estimation of STromal and Immune cells in MAlignant Tumor tissues using Expression data (ESTIMATE) algorithm was employed to quantify the stromal score, immune score, estimated score, and tumor purity for each NPC sample (16, 17). Next, we used the CIBERSORT (18), EPIC (19), and quanTIseq (20) algorithms to characterize TME composition in the NPC samples. We also explored the association between KIF18B and immune-related gene expression (21).

### 2.6 Evaluating the association between KIF18B and TME features at the single-cell level

We obtained scRNA-seq data for NPC samples from the GEO database (https://www.ncbi.nlm.nih.gov/geo/; accession number: GSE150430) and CNGB Nucleotide Sequence Archive (https://db.cngb.org/; accession code: CNP0000428). This dataset comprised 48,584 single cells from 15 primary NPC tumors and one normal samples. The Seurat R package was utilized to analyze scRNA-seq data (22). Cells underwent quality filtering to remove those containing fewer than 200 or more than 9,000 expressed genes, or having more than 20% of unique molecular identifiers derived from the mitochondrial genome. For the remaining cells, the “ScaleData” function in Seurat package was used to log normalized the count. The analysis of t-distributed stochastic neighbor embedding (tSNE) dimensionality reduction and cell annotations were directly derived from information provided in the dataset. Next, we used the BisqueRNA package to construct a reference basis matrix of expression profiles for individual cell types from the GSE150430 dataset and estimated cell proportions from bulk expression (i.e., GSE102349 dataset) (23). We stratified the patients from GSE150430 cohort into KIF18B-high and-low groups using the “AverageExpression” function. Briefly, we calculated the average expression of KIF18B across all cells in each patient. Then, we extracted regulatory T cell (Treg) subpopulations from each patient and aggregated them based on the previously calculated average KIF18B expression. Gene profiling and biological analysis via GSEA were also performed on Treg subpopulations from KIF18B-low and -high groups. CellChat is an open source R tool that infers and analyzes cell-cell communication networks using scRNA-seq and spatial transcriptomic data (24). We examined the communication patterns between different compartments using CellChat package. Significant interactions (i.e., ligand-receptor pairs) between some cell groups and other cell groups (*P* < 0.01) are displayed.

### 2.7 Immunofluorescence assays

The 29 NPC samples in our institutional cohort were divided into low and high expression groups based on the median value of IHC score of KIF18B. Eight samples were randomly selected from each group and conducted immunofluorescence staining. Briefly, the steps before incubation of secondary antibody were same as described in the above IHC protocol. The following primary antibodies were used: anti-FOXP3 (1:200, 98377S, Cell Signaling Technology), anti-CD8 (1:100, ZA-0508, OriGene), and anti-IBA-1 (1:200, 17198, Cell Signaling Technology). After incubation of primary antibodies, the slides were incubated with DyLight 488-labeled secondary antibody (1:500, ab96899, Abcam) in the dark at 37°C for 1h. Images were captured using immunofluorescence microscopy (Olympus, Tokyo, Japan). The quantification of cell numbers was carried out manually by counting the positive cells, while the immunofluorescence area was automatically identified using Image Pro Plus software (version 6.0.0.260).

### 2.8 Pseudotime trajectory analysis

To elucidate the developmental trajectory of NPC cells, a subcluster analysis was conducted on malignant cells. The “FindVariableFeatures” function in Seurat package was employed to identify 2,000 highly variable genes. Moreover, a tSNE method was applied for non-linear dimensionality reduction. The number of principal components utilized was determined through an elbow plot coupled with an exploration of top genes associated with each principal component. The “FindClusters” function was employed for cell clustering by graph-based clustering approach. Next, we conducted pseudotime analysis using the Monocle2 R package (25). The “dispersionTable” function in Monocle2 was used to identify the DEGs along pseudotime.

### 2.9 Prediction of m6A modification site

We retrieved the sequences of KIF18B transcripts from the NCBI website (https://www.ncbi.nlm.nih.gov/) and predicted their m6A modification sites and local structures using the SRAMP website (http://www.cuilab.cn/sramp).

### 2.10 Prediction of the efficacy of immunotherapy and chemotherapy

To predict the response to immunotherapy, we applied the Tumor Immune Dysfunction and Exclusion (TIDE) algorithm, according to the instructions on the TIDE website (26, 27) (http://tide.dfci.harvard.edu/). The TIDE, merk18 signature, and T cell exclusion were obtained from the output results. These scores indicate the likelihood of immune escape, with higher TIDE scores suggesting greater resistance. Merck18 signature reflects the T cell-based inflammation status of the tumors, which can be classified as “hot” (T cell inflamed) or “cold” (T cell non-inflamed) based on the presence of immune cells (28). Moreover, we calculated the immunophenoscore (IPS) to evaluate immunotherapy sensitivity from an immunogenic perspective (29). A high IPS is generally associated with a better response to immune checkpoint inhibitors. Lastly, we used the R package OncoPredict to estimate the IC50 values of several drugs in NPC patients with different levels of KIF18B expression (30). The expression matrix and drug treatment information of the training set were derived from the Genomics of Drug Sensitivity in Cancer dataset version.2 (https://www.cancerrxgene.org/) (31).

### 2.11 Statistic analysis

Statistical analysis and visualization were performed using GraphPad Prism 8.0 and R software (version 4.2.2). The R packages used in this study were listed as followed: survival, survminer, rms, timeROC, limma, ComplexHeatmap, clusterProfiler, IOBR, msigdbr, Seurat, BisqueRNA, CellChat, Monocle2, OncoPredict, car, stats, ggplot2, stringr, dplyr, future, future.apply, data.table, and VennDiagram. The t-test or Wilcoxon rank-sum test was used to compare two independent groups of numerical variables, while one-way ANOVA or Kruskal-Wallis test was applied to examine the two groups. The chi-square test, Yates correction, or Fisher’s exact test was used to analyze categorical variables. The log-rank test and Cox regression model were used for survival analysis. Factors with a *P*-value ≤ 0.1 in the univariate analysis were included in the subsequent multivariate analysis. This approach was adopted due to a higher α level of significance is considered appropriate to mitigate the potential exclusion of relevant independent covariates. Pearson and Spearman correlation analyses were used to assess the correlation between continuous and ordinal factors, respectively. *P* < 0.05 (two-tailed) was set as the significance level for all analyses, unless otherwise specified.

## 3 Results

### 3.1 KIF18B is elevated in NPC and indicates worse survival

**Figure 2:**
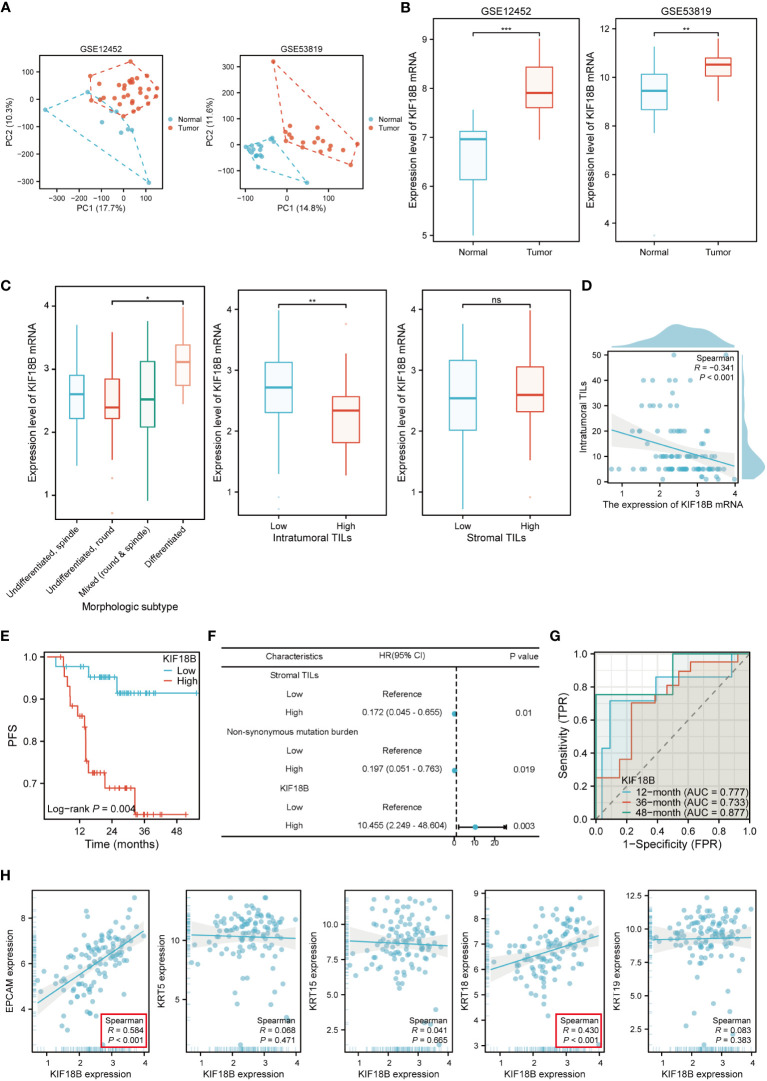
KIF18B mRNA is highly expressed in NPC and is associated with poor prognosis. **(A)** The principal component analysis of GSE12452 and GSE53819. **(B)** KIF18B mRNA expression level in two datasets (t-test). **(C)** KIF18B mRNA expression level in NPC patients with different morphologic subtypes (one-way ANOVA), intratumoral TILs scores (Wilcoxon rank-sum test) and stromal TILs scores (t-test). **(D)** The correlation between KIF18B mRNA expression level and intratumoral TILs scores. **(E)** The Kaplan-Meier survival analysis of PFS for NPC patients with different KIF18B expression in GSE102349 dataset (low *n* = 44, high *n* =44). **(F)** Multivariate cox regression analysis of PFS. **(G)** The diagnostic power of KIF18B mRNA for NPC patients along time. **(H)** Correlation analysis of KIF18B and classic NPC markers. *P < 0.05, **P < 0.01, ***P < 0.001; ns, not significant; PC, principal component; TILs, tumor-infiltrating lymphocytes; PFS, progression-free survival; HR, hazard ratio; CI, confidence interval; AUC, area under curve; NPC, nasopharyngeal carcinoma.

The mRNA expression of KIF18B in NPC tissues was upregulated compared to that in normal nasopharyngeal samples (**Figures 2A, B**; **Supplementary Figure S1**). Histopathological analysis showed noticeable variations in the levels of KIF18B expression among NPC patients with different morphological subtypes and intra-tumoral tumor-infiltrating lymphocytes (TILs) scores, but not in stromal TILs scores (**Figure 2C**). Furthermore, the expression level of KIF18B was significantly negatively correlated with intra-tumoral TILs scores (**Figure 2D**). In Cox regression analysis, KIF18B was identified as an independent adverse prognostic factor, with high predictive efficacy for PFS (**Figures 2E-G**; **Supplementary Table S6**). We further observed that KIF18B was positively correlated with classic malignant or epithelial markers (32, 33), such as EPCAM and KRT18 (**Figure 2H**).

**Figure 3:**
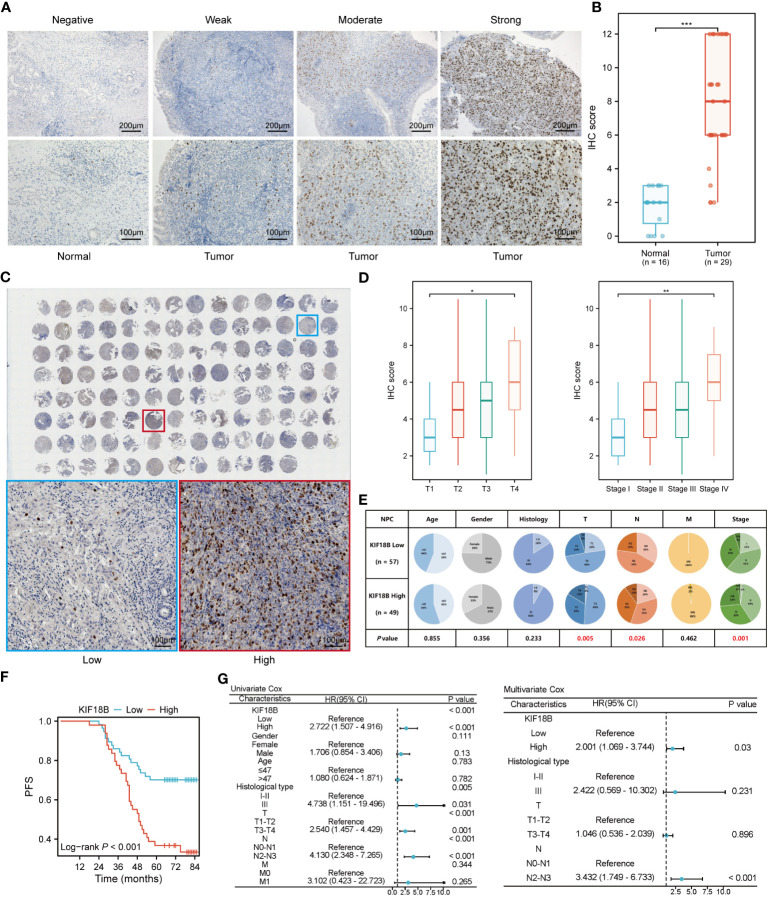
KIF18B protein is elevated in NPC and indicates worse survival. **(A)** Representative IHC staining images of KIF18B in NPC and non-malignant tissues. **(B)** The statistical analysis of KIF18B IHC score in tumor and non-malignant tissues (Wilcoxon rank-sum test). **(C)** IHC staining for KIF18B in 110 clinical NPC samples from tissue microarray (upper). The images on the lower are representative images. **(D)** IHC score of KIF18B in NPC patients with different T and stage classifications (Kruskal-Wallis test). **(E)** The proportion difference of clinical indices in the KIF18B high and low expression groups from the tissue microarray cohort (chi-square test for differential analysis of age, gender, histological type and T classification; Yates correction for differential analysis of N classification; Fisher’s exact test for differential analysis of M classification). **(F)** The Kaplan-Meier survival analysis of PFS in NPC patients (low *n* = 57, high *n* =49). **(G)** Cox regression analysis of PFS. *P < 0.05, **P < 0.01, ***P < 0.001; IHC, immunohistochemistry; PFS, progression-free survival; HR, hazard ratio; CI, confidence interval; NPC, nasopharyngeal carcinoma.

To confirm KIF18B protein expression, we performed IHC assays on our institutional cohort. Consistent with the mRNA expression results, we observed high KIF18B protein expression in NPC samples, mainly in the nucleus (**Figures 3A, B**). The prognostic and diagnostic value of KIF18B in NPC was also evaluated using tissue microarray (**Figure 3C**). Patients with higher KIF18B expression showed more advanced T classification, N classification, and stage (**Figures 3D, E**) and had worse overall survival (OS), distant metastasis-free survival (DMFS), PFS, and relapse-free survival (RFS) (**Figure 3F**; **Supplementary Figure S2**). Cox regression analysis revealed that high KIF18B expression was associated with an unfavorable prognosis (**Figure 3G**). Furthermore, we also observed that the prognostic performance of KIF18B strengthens over time (**Supplementary Figure S3**). Nonetheless, given the relatively modest sample size and the limitations inherent to semi-quantitative analysis, there is a need for a multicenter study with an expanded sample size, integration of other potential factors and incorporation of artificial intelligence-driven digital pathology techniques (34). This comprehensive approach aims to enhance our ability to assess the predictive potential of KIF18B concerning the survival outcomes in NPC.

### 3.2 KIF18B promotes EMT and regulates immune-related pathways

**Figure 4:**
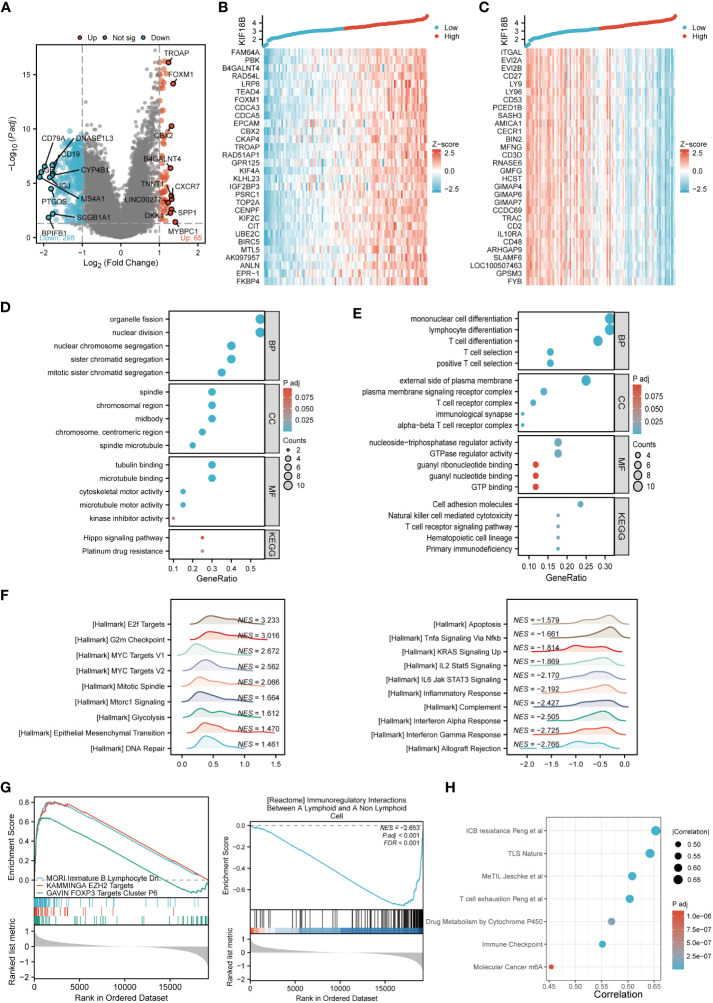
Comprehensive functional enrichment analysis of KIF18B in NPC. **(A)** Volcano map of DEGs between KIF18B high and low expression groups. **(B)** Heat map of top 30 positive co-expression DEGs with KIF18B. **(C)** Heat map of top 30 negative co-expression DEGs with KIF18B. The GO and KEGG analysis for genes belonging to the co-expression DEGs with strong positive **(D)** and negative correlation **(E)** with KIF18B. **(F)** The enrichment score of hallmark pathways influenced by KIF18B. **(G)** The enrichment score of curated gene sets influenced by KIF18B. **(H)** The bubble plot illustrated the signature gene sets that were highly associated with KIF18B expression and exhibited significant differential expression between the high and low KIF18B expression groups. BP, biological process; CC, cellular component; MF, molecular function; KEGG, Kyoto Encyclopedia of Genes and Genomes; NPC, nasopharyngeal carcinoma; DEGs, differential expression genes; GO, gene ontology; NES, normalized enrichment score; ICB, immune checkpoint blockade; TLS, tertiary lymphoid structures; MeTIL, methylation of tumor-infiltrating lymphocytes; m6A, N6-methyladenosine.

To elucidate the biological functions of KIF18B, we determined DEGs between the high- and low- KIF18B expression groups; the top 30 DEGs that showed positive or negative correlation with KIF18B were shown (**Figures 4A-C**). GO and KEGG analysis showed that the genes positively correlated with KIF18B (R > 0.7) were involved in cell division and platinum drug resistance (**Figure 4D**). Conversely, genes negatively correlated with KIF18B (R < -0.7) in NPC samples were enriched in various immune-related pathways (**Figure 4E**).

To further characterize potential functions of KIF18B, GSEA was conducted on gene sets derived from MSigDB collections. The results revealed that heightened KIF18B expression correlated with the elevation of gene sets associated with the cell cycle (e.g., G2M checkpoint), oncogenic pathways (e.g., MYC targets), cell division (e.g., mitotic spindle), cell growth (e.g., mTORC1 signaling), glycolysis, EMT, DNA repair, and immunosuppression (e.g., FOXP3 and EZH2 targets) (**Figures 4F, G**). By contrast, the low KIF18B expression group showed enrichment of genes implicated in innate and adaptive immune activation (e.g., TNFα signaling via NFκB, IL2/STAT5 signaling, IL6/JAK/STAT3 signaling, inflammatory response, complement, and interferon α and γ response), immunoregulatory interactions between lymphoid and non-lymphoid cells, and apoptosis (**Figures 4F, G**). We analyzed the popular signature gene sets integrated using the IOBR package, and found that KIF18B was correlated with m6A (35), tertiary lymphoid structures (36), and other immune-related signatures (**Figure 4H**).

### 3.3 High expression of KIF18B is related to impaired anti-tumor immunity

**Figure 5:**
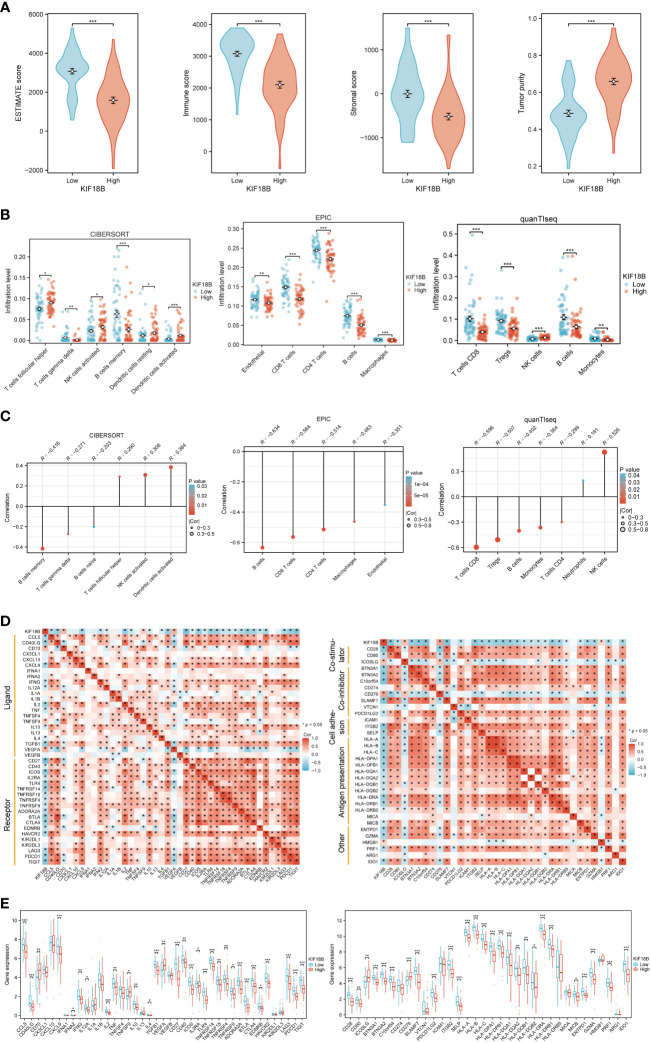
The immunosuppressive function of KIF18B in NPC. **(A)** Comparison of ESTIMATE score, immune score, stromal score and tumor purity calculated by ESTIMATE algorithm (t-test for differential analysis of ESTIMATE score and tumor purity; Wilcoxon rank-sum test for immune and stromal score). **(B)** The immune cell types with statistical differences in infiltration level between the high and low expression groups of KIF18B were shown (Wilcoxon rank-sum test). **(C)** The immune cell types with significant correlation with KIF18B expression were showed. **(D)** Relevance of expression between KIF18B and immune-related genes. **(E)** Differential expression of immune-related genes in different KIF18B expression groups (Wilcoxon rank-sum test). *P < 0.05, **P < 0.01, ***P < 0.001; ESTIMATE, Estimation of STromal and Immune cells in MAlignant Tumor tissues using Expression data; NK cells, natural killer cells; Tregs, regulatory T cells; NPC, nasopharyngeal carcinoma.

Functional enrichment analysis showed that KIF18B plays a vital role in immune regulation; therefore, we further explored the potential functions of KIF18B in the tumor immune microenvironment. ESTIMATE analysis revealed a general decrease in infiltration by both immune and stromal cells under high KIF18B expression (**Figure 5A**). The analysis of CIBERSORT, EPIC and quanTIseq algorithms revealed that high KIF18B expression was associated with reduced infiltration by CD8^+^ T cells, CD4^+^ T cells, γδT cells, B cells, macrophages, and monocytes, and increased infiltration by natural killer (NK) cells and dendritic cells (**Figures 5B, C**). In addition, we examined the expression of tumor immunity-related genes and found that most were negatively correlated with KIF18B expression (**Figures 5D, E**).

### 3.4 KIF18B expression is strongly positively correlated with activated regulatory T cells

**Figure 6:**
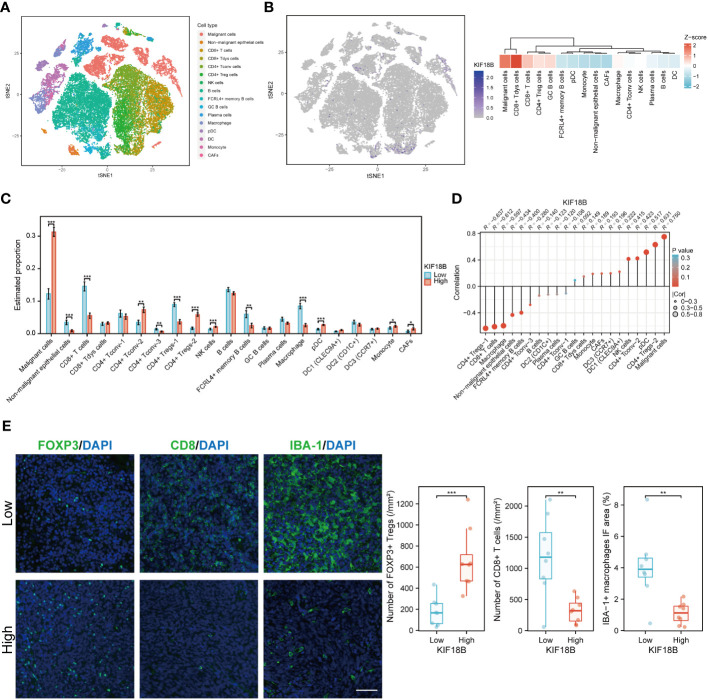
The scRNA-seq analysis reveals the expression of KIF18B in different cell types within NPC tumor microenvironment and its association with diverse cell types. **(A)** Single-cell atlas of all cells in GSE150430 dataset. **(B)** The expression and distribution of KIF18B in GSE150430 dataset. **(C)** The proportion of different cell types in the samples of the GSE102349 dataset estimated by the deconvolution algorithm based on NPC scRNA-seq data (Wilcoxon rank-sum test). **(D)** Correlation analysis between KIF18B and these cell types. **(E)** Left, representative immunofluorescence images for FOXP3, CD8 and IBA-1 in NPC tumor samples with low (upper) and high (lower) KIF18B expression. Right, quantitation of the number of FOXP3^+^ Tregs and CD8^+^ T cells and the percentage of IBA-1^+^ macrophage immunofluorescence area (low *n* = 8, high *n* = 8, t-test). *P < 0.05, **P < 0.01, ***P < 0.001; tSNE, t-distributed stochastic neighbor embedding; CD8+ Tdys cells, dysfunctional CD8^+^ T cells; CD4+ Tconv cells, conventional CD4^+^ T cells; CD4^+^ Treg cells, regulatory CD4^+^ T cells; NK, natural killer; GC, germinal center; pDC, plasmacytoid dendritic cell; DC, dendritic cell; CAFs, cancer-associated fibroblasts; Tregs, regulatory T cells; IF, immunofluorescence; scRNA-seq, single-cell RNA sequencing; NPC, nasopharyngeal carcinoma. Scale bar: 100 µm.

At the single-cell level, KIF18B was mainly expressed in malignant and dysfunctional CD8^+^ T cells (**Figures 6A, B**). Using a deconvolution method based on the NPC scRNA-seq data, we estimated the cell composition in bulk expression and examined the association between KIF18B and diverse cells in TME, which Chen et al., had previously classified into 21 subclusters according to their features (33). Consistent with previous results (**Figures 5B, C**), we observed that the infiltration of CD8^+^ T cells and macrophages in the KIF18B-high expression group was significantly lower than that in the low-expression group, whereas the infiltration by NK cells and plasmacytoid dendritic cells was markedly higher in the KIF18B-high group (**Figure 6C**). It was noticed that Tregs exhibited heterogeneity; Chen et al., divided CD4^+^ Tregs into CD4^+^ Tregs-1 and CD4^+^ Tregs-2 (33). The co-stimulatory molecules were highly expressed in CD4^+^ Tregs-2 (33), suggesting that they may represent an activated suppressive Tregs cluster, whereas CD4^+^ Tregs-1 are likely to be resting cells. Infiltration level of CD4^+^ Tregs-2 in the KIF18B-high group was significantly higher than that in the low-expression group (**Figure 6C**). Among the 19 subclusters of immune cells, CD4^+^ Tregs-2 exhibited a robust positive correlation with the expression of KIF18B, as shown by the highest correlation coefficient (R = 0.631). By contrast, CD8^+^ T cells and macrophages showed a remarkably negative correlation with KIF18B expression, as shown by correlation coefficients of -0.612 and -0.597, respectively (**Figure 6D**; **Supplementary Figure S4**). The change of major immune cell infiltration with KIF18B expression in NPC was then confirmed by immunofluorescence analysis (**Figure 6E**). Compared to the low KIF18B expression group, the high KIF18B expression group displayed a notable rise in Foxp3^+^ Tregs infiltration, accompanied by a substantial decrease in the infiltration of CD8^+^ T cells and macrophages.

The gene profiles of Treg subpopulations were investigated from KIF18B-low and -high groups in scRNA-seq samples (**Supplementary Figure S5A**). Naive markers exhibited decreased expression in Tregs from the KIF18B-high group. Additionally, Tregs within the KIF18B-high group displayed heightened expression of co-stimulatory molecules and an increased propensity for proliferation. Notably, the secretion of anti-inflammatory factors, such as CCL18 and IL10, was also augmented. Biological analysis uncovered that Tregs from the KIF18B-high group demonstrated enrichment in pathways associated with cell proliferation (e.g., E2F targets), activation (e.g., MYC, IFNG and TGFB1 signaling), chemotherapy resistance, facilitation of EMT development, and suppressive immune microenvironment (**Supplementary Figure S5B**).

Moreover, we found that there was no significant correlation between the tumor mutational burden (TMB) and the infiltration level of most immune cells, and that the PFS outcome did not significantly differ between different TMB levels (**Supplementary Figure S6**), suggesting that TMB might not be an effective predictor of immunotherapy response in NPC.

### 3.5 KIF18B-high malignant cells modulate immunosuppressive cell-cell interactions in the TME

**Figure 7:**
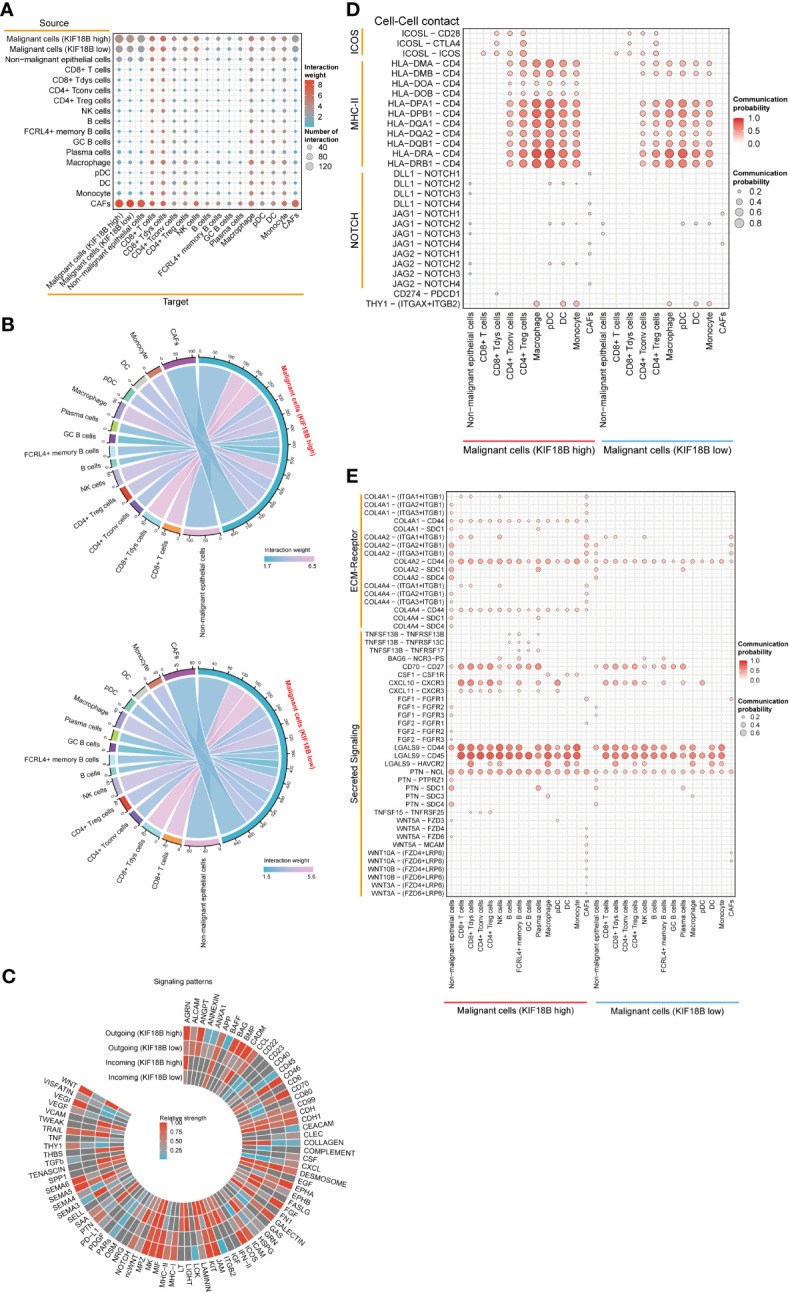
The effects of KIF18B on intercellular interactions. **(A)** Bubble plot showing the number of interaction and interaction weight between all types of cells predicted by CellChat. **(B)** Detailed view of the ligands expressed by malignant cells with different expression of KIF18B and the cells expressing the cognate receptors primed to receive the signal. Numbers and line thickness indicate the quantity of ligand-receptor pairs for each intercellular link. **(C)** The relative strength of the outgoing and incoming signal patterns of malignant cells with different expression of KIF18B. Overview of selected ligand-receptor interactions of **(D)** cell-cell contact, **(E)** ECM-receptor and secreting signaling between high/low KIF18B expression malignant cells and other cells within tumor environment. CD8+ Tdys cells, dysfunctional CD8^+^ T cells; CD4+ Tconv cells, conventional CD4^+^ T cells; CD4+ Treg cells, regulatory CD4^+^ T cells; NK, natural killer; GC, germinal center; pDC, plasmacytoid dendritic cell; DC, dendritic cell; CAFs, cancer-associated fibroblasts; ECM, extracellular matrix.

Using CellChat, we constructed an intercellular communication network among the component cells of the TME (**Figure 7A**). We found that malignant cells showing high KIF18B expression had more extensive communication and a higher interaction weight with other cells, than those with low KIF18B expression (**Figures 7A, B**). Notably, the overall signal patterns demonstrated that malignant cells with high KIF18B expression triggered more immunosuppressive pathways, such as the BAG, NOTCH, PTN, SAA, SEMA5/6, and WNT, suggesting that these molecular interactions were involved in the impaired anti-tumor immunity mediated by KIF18B (**Figure 7C**). Next, we characterized the ligand-receptor pairs in malignant cells with different KIF18B expression levels and immune cells (**Figures 7D, E**). Enhanced stimulatory interactions between KIF18B-high malignant cells and CD4^+^ Tregs were commonly observed. In addition to increased immunomodulatory cytokine-receptor pairs (e.g., ICOSL-ICOS, TNFSF15-TNFRSF25, and PTN-NCL), higher levels of chemokine (e.g., CXCL10/11-CXCR3) and extracellular matrix (e.g., COL4A2/4-CD44) signaling pathways were also detected. Moreover, we found higher expression of immune checkpoint molecules, such as cytotoxic T lymphocyte-associated protein 4 (CTLA-4) and HAVCR2, in CD4^+^ Tregs. We also identified that putative communications, such as inhibitory interactions mediated by CD274-PDCD1, may contribute to the exhaustion of CD8^+^ T cells induced by malignant cells highly expressing KIF18B (**Figure 7D**).

### 3.6 KIF18B expression increases along the pseudotime trajectory and promotes EMT in malignant cells

**Figure 8:**
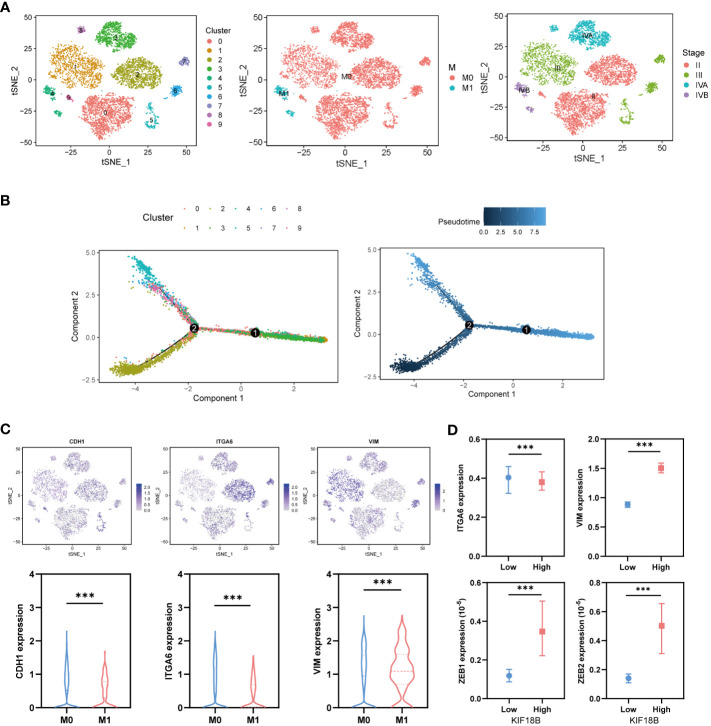
KIF18B is upregulated in the advanced malignant cell and enhances epithelial-mesenchymal transition. **(A)** Using tSNE algorithm to reduce the dimensionality of malignant cells into ten clusters. **(B)** Pseudo-trajectory and cell source transition of malignant cells. **(C)** The expression of CDH1, ITGA6, and VIM in malignant cells derived from metastatic and non-metastatic samples (Wilcoxon rank sum test). **(D)** Comparison of the ITGA6, VIM, ZEB1 and ZEB2 expression between malignant cells with high and low expression of KIF18B (Wilcoxon rank sum test). ***P < 0.001; tSNE, t-distributed stochastic neighbor embedding.

To explore the developmental process of malignant NPC cells, we first clustered them into ten different subgroups (**Figure 8A**). We then simulated the motion trajectories of malignant cells from M0 and M1 samples and established a tree-like structure of the entire pedigree differentiation trajectory to compare the states of malignant cells. The cells at the base of the tree-like structure were mostly non-metastatic malignant cells, whereas the metastatic malignant cells tended to be concentrated in more advanced states (**Figure 8B**). Notably, the expression of KIF18B in malignant cells showed an upregulation trend along the pseudotime trajectory (**Supplementary Figure S7**), suggesting its role in metastasis. By analyzing the expression of E-cadherin (CDH1), ITGA6, and vimentin (VIM) (**Figure 8C**), we found that type III EMT may be involved in the metastasis of malignant NPC cells. Moreover, the upregulation of KIF18B was related to decreased ITGA6 and increased VIM, ZEB1, and ZEB2 expression (**Figure 8D**), thereby corroborating the role of KIF18B in promoting EMT in functional enrichment analysis (**Figure 4F**).

### 3.7 KIF18B is highly correlated with m6A-related genes

**Figure 9:**
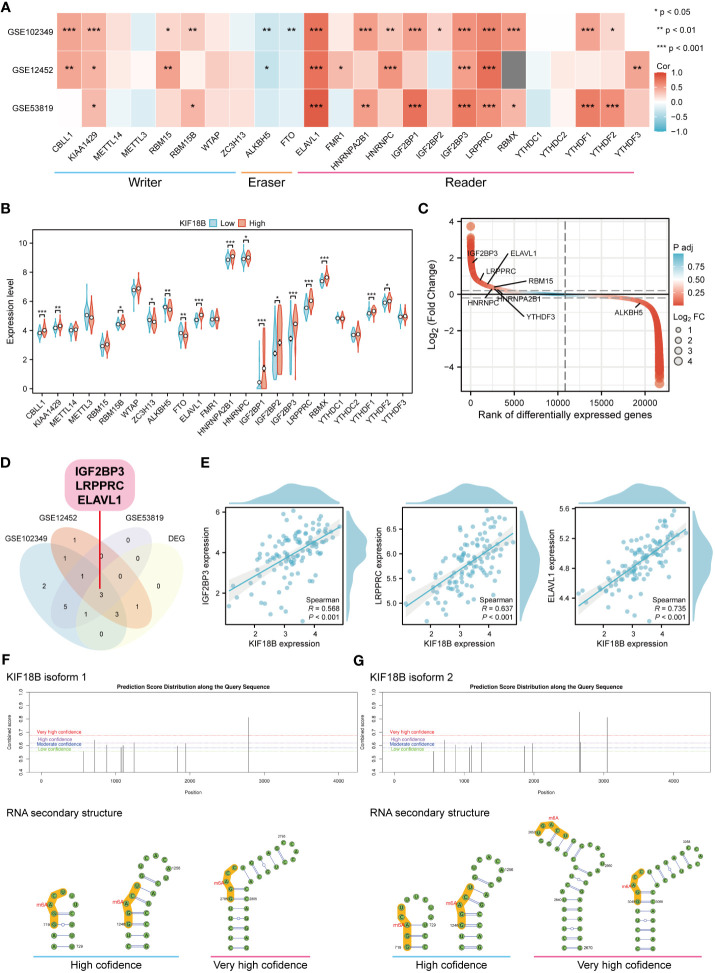
KIF18B is highly correlated with m6A-related genes. **(A)** Heat map of the correlation between KIF18B and m6A-related gene expression. **(B)** Differential expression of m6A-related gene between KIF18B high and low expression groups. **(C)** Differential expression genes between NPC and non-malignant nasopharyngeal tissues. The horizontal dashed lines represent log_2_(Fold change) equal to 0.2 and -0.2, respectively. The m6A-related genes with |log_2_(Fold change)| > 0.3 and adjust *P*-value < 0.05 were labeled. **(D)** Venn diagram of m6A-related genes significantly associated with KIF18B and DEGs in NPC. **(E)** The scatter plot shows the correlation between the overlapping genes and KIF18B. **(F, G)** Potential m6A methylation sites and secondary structures of KIF18B mRNA are illustrated schematically. *P < 0.05, **P < 0.01, ***P < 0.001; m6A, N6-methyladenosine; DEGs, differential expression genes; NPC, nasopharyngeal carcinoma.

Epigenetic modifications are crucial for the pathogenesis and progression of malignant tumors (37, 38). In this study, functional enrichment analysis uncovered the connection between KIF18B and m6A methylation (**Figure 4H**), one of the most prevalent RNA modification processes (39). By analyzing three public datasets, we were able to verify the association between KIF18B and most m6A-related genes (**Figure 9A**). Furthermore, we observed that, compared to the KIF18B-low expression group, the KIF18B-high expression group exhibited elevated expression of most m6A “writer” and “reader” genes, and reduced expression of m6A “eraser” genes (**Figure 9B**). Differential analysis was performed to identify the m6A-related genes that were significantly upregulated or downregulated in NPC (**Figure 9C**). We identified three genes that were shared by the three GEO datasets and DEGs (**Figure 9D**). These genes exhibited strong positive correlations with KIF18B expression (**Figure 9E**). The Kaplan-Meier survival curve indicated that ELAVL1 was the only prognostic factor (**Supplementary Figure S8**). During the prediction of m6A distribution and secondary structures for two KIF18B isoforms, we detected a relatively high number of m6A sites in KIF18B mRNA (**Figures 9F, G**; **Supplementary Tables S7** and **S8**).

### 3.8 KIF18B acts as a predictor for immunotherapy and chemotherapy response

**Figure 10:**
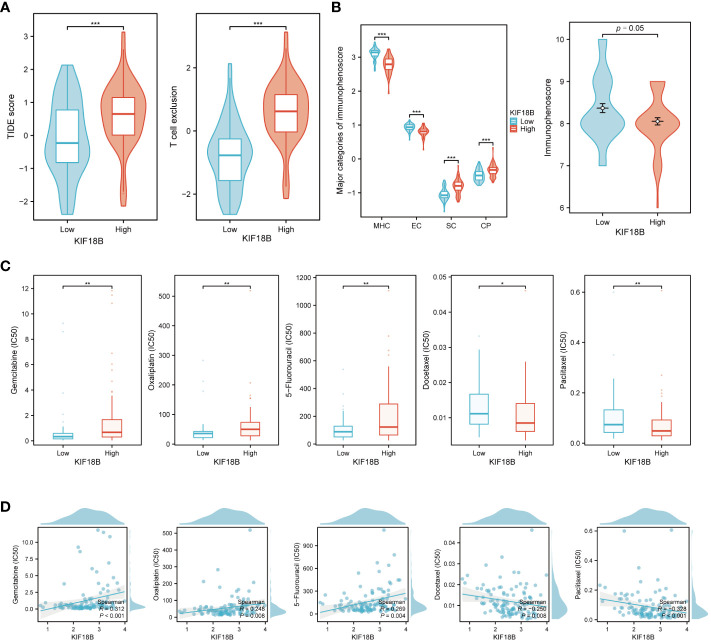
KIF18B serves as a predictive biomarker for both immunotherapy and chemotherapy response. **(A)** Comparison of TIDE score and T cell exclusion score between KIF18B-high and KIF18B-low group (t-test). **(B)** Comparison of immunophenoscore between KIF18B-high and KIF18B-low group (Wilcoxon rank-sum test). **(C)** Comparison of the IC50 values of common chemotherapy drugs between different KIF18B expression groups (Wilcoxon rank-sum test). **(D)** Correlation analysis between KIF18B expression and IC50 values of these chemotherapy drugs. *P < 0.05, **P < 0.01, ***P < 0.001; TIDE, Tumor Immune Dysfunction and Exclusion; MHC, major histocompatibility complex; EC, effector cells; SC, immunosuppressive cells; CP, immune checkpoints.

**Figure 11:**
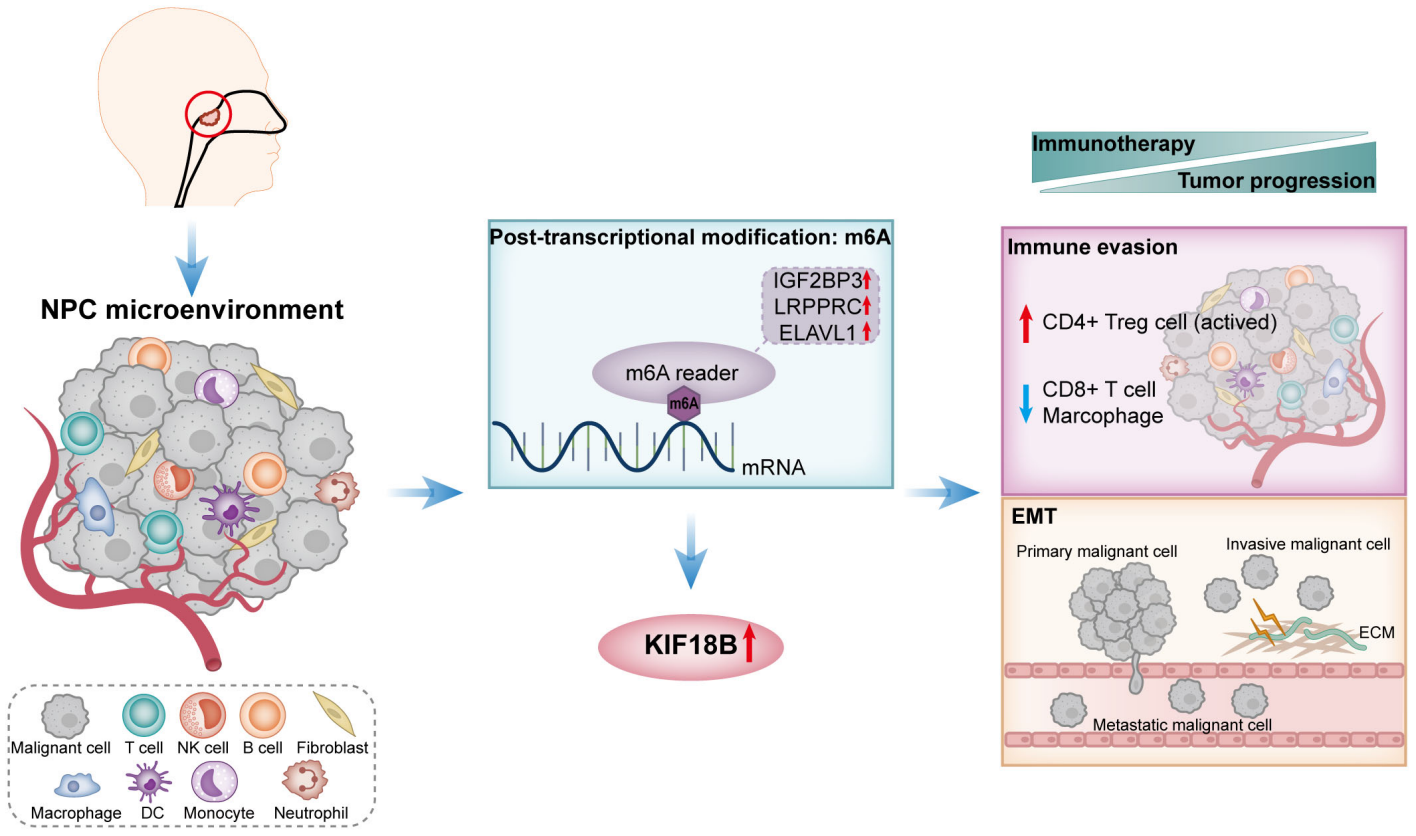
A schematic diagram of the mechanism summary elucidated in this study. In NPC, KIF18B exhibits upregulated expression, potentially attributed to m6A modification. The elevated expression of KIF18B influences immune escape through mechanisms such as promoting the expansion of activated CD4^+^ Treg cells, while simultaneously reducing the proportion of CD8^+^ T cells and macrophages. Additionally, KIF18B plays a regulatory role in EMT-related pathways. Therefore, KIF18B contributes to tumor progression and immunotherapy resistance. NPC, nasopharyngeal carcinoma; NK, natural killer; DC, dendritic cell; m6A, N6-methyladenosine; CD4+ Treg cell, regulatory CD4^+^ T cells; EMT, epithelial-mesenchymal transition; ECM, extracellular matrix.

According to the TIDE algorithm, the KIF18B-high group showed significantly higher TIDE and T cell exclusion scores as well as lower Merck18 signature scores (**Figure 10A**; **Supplementary Figure S9**). The KIF18B-high group also showed a marginally lower IPS than the KIF18B-low group (*P* = 0.05; **Figure 10B**). These metrics indicated that higher KIF18B expression was more likely to generate immune escape and immunotherapy resistance. Previous studies have reported that KIF18B knockdown may increase the sensitivity of colon and breast cancer cells to oxaliplatin (40) and doxorubicin (41), respectively. Furthermore, colorectal cancer cells can enhance oxaliplatin and 5-fluorouracil resistance by recruiting Treg or promoting its expansion (42, 43), while the significance of Tregs in conferring chemoresistance in ovarian cancer has also been emphasized (44). Consistently, we found that NPC patients with high KIF18B expression had significantly higher IC50 values for gemcitabine, oxaliplatin and 5-fluorouracil than those with low KIF18B expression (**Figures 10C, D**). By contrast, the IC50 values of docetaxel and paclitaxel in the KIF18B high-expression group were lower (**Figures 10C, D**). These findings aligned with the aforementioned enrichment analysis that Tregs from the KIF18B-high group were linked to resistance against fluorouracil and alkylating agents (**Supplementary Figure S5B**). In summary, high KIF18B expression may indicate tumor progression and poor immunotherapy outcomes (**Figure 11**).

## 4 Discussion

Kinesin superfamily proteins (KIFs) are a group of proteins that share highly conserved motor domains and participate in numerous biological processes such as mitosis, vesicle trafficking, and maintenance of cell polarity, which are essential for the morphology and function of cells (45, 46). Recent studies have revealed that many KIFs are aberrantly expressed in different tumor cells, where they play oncogenic roles (47–49). However, the role of KIF18B in NPC has remained unclear. In this study, our bioinformatics analyses of public datasets indicated that KIF18B was significantly overexpressed in NPC tissues, which was confirmed by IHC arrays conducted using NPC tissues from our institutional cohort. We also found that KIF18B expression was associated with advanced T, N, and stage classifications in patients with NPC. Furthermore, high KIF18B expression was an independent predictor of poor prognosis and a reliable diagnostic marker of recurrence and metastasis.

Recently developed tumor-specific immunotherapeutic strategies, based on the mechanism of immune evasion, reportedly present a promising approach for NPC therapy (50). KIF18B has been implicated in the immune infiltration of various tumors, including head and neck squamous cell carcinoma (HNSCC) (10). However, its role in HNSCC differed from that revealed by our findings, implying that KIF18B may exert different effects on tumor-related immunity in NPC and HNSCC. Functional enrichment analyses have collectively indicated that KIF18B may exert immunobiological functions in NPC tumorigenesis. Our scRNA-seq analysis detected KIF18B expression in malignant cells as well as in other cells, such as dysfunctional CD8^+^ T cells and germinal center B cells, implying that KIF18B is closely associated with the TME and may exert its effect on multiple cell types. The overall analysis of immune infiltration revealed that the high KIF18B expression group had an immunosuppressive TME, as evidenced by lower levels of stromal and immune cells.

To assess the association between KIF18B expression and the abundance of specific immune cells, we applied three algorithms that rely on the common gene expression signatures of immune cell subtypes, as well as a deconvolution algorithm that used NPC scRNA-seq data to estimate the infiltration levels of different immune cells in samples. The four algorithms yielded generally consistent results, except for monocytes. This reflected the distinctiveness of the gene expression profiles of monocytes within the TME of NPC. However, a previous study reported that monocytes may not have a reliable prognostic value in NPC, suggesting that KIF18B does not affect prognosis by modulating monocyte (33). NK cells facilitate the immune response against tumors by killing target cells and producing cytokines (51). However, NK cells also actively participate in the resistance to microbial infection (51). NPC being an Epstein-Barr virus (EBV)-related tumor, EBV infection may lead to NK cell proliferation in NPC. In this study, increased KIF18B expression was associated with higher tumor cell purity, which may possibly clarify the positive correlation seen between KIF18B expression and NK cell infiltration.

Penetration of the tumor parenchyma by lymphocytes, especially CD8^+^ T cells, is essential for anti-tumor immunity, which forms the basis of immunotherapeutic effects (52, 53). Previous evidence showed that KIF18B was positively correlated with CD8^+^ T cells in renal clear cell carcinoma and lung carcinoma (10). Conversely, we observed a strong negative association between CD8^+^ T cells and KIF18B expression (R = -0.612), which were confirmed by immunofluorescence assay. Besides, the anti-tumor immunity conferred by CD8^+^ T cell contributed to tumor regression and improved prognosis in NPC patients (54, 55), which in line with our finding that low KIF18B expression group exhibited better prognosis. Tumor-associated macrophages (TAMs) are macrophages that infiltrate or accumulate in the TME, where they secrete various cytokines and chemokines, and act as regulators of anti-tumor immunotherapy (56). TAMs are highly expressed in NPC and associated with favorable clinical outcomes (33). In present study, the high abundance of TAMs in NPC and its negative association with KIF18B was confirmed by immunofluorescence analysis.

Tregs, which are essential for maintaining immune self-tolerance and homeostasis, abound in various cancers (57, 58). Recent research suggests that within NPC patients facing an unfavorable prognosis, there is a noteworthy elevation in Tregs density. Moreover, their engagement is particularly pronounced in the proximity of tumor cells and cytotoxic T lymphocytes, and they even co-localize with cytotoxic T lymphocytes (59). Drawing from a multicenter single-cell cohort study, it has come to light that NPC cells can heighten the lipid-driven development, specialized functionality, and homeostasis of Tregs through the CD70-CD27 interaction, ultimately resulting in immune evasion (60). Besides, as opposed to the gene expression profiles of all Tregs, activated Tregs exhibit gene signatures linked to poor prognosis (61). Of the immune cells included in this study, activated Tregs correlated the most with KIF18B expression (R = 0.631). *In vitro*, we confirmed a heightened infiltration of Tregs in the high KIF18B expression group. Similarly, in prostate adenocarcinoma and renal clear cell carcinoma, highly expressed KIF18B exhibited a significant positive correlation with Tregs, albeit with relatively low correlation coefficients of 0.18 and 0.29, respectively (10). In contrast, KIF18B demonstrated a significant negative correlation with Tregs in skin cutaneous melanoma (R = -0.19), lymphoid neoplasm diffuse large B-cell lymphoma (R = -0.51), and bladder urothelial carcinoma (R = -0.26) (10). Thus, abnormal infiltration and activation of Tregs associated with KIF18B may be the key and unique immune mechanism underlying NPC progression.

KIF18B may regulate the cytokines or chemokines that recruit and activate CD4^+^ Tregs. In cell-cell communication analysis, ICOSL-CD28/ICOS and TNFSF15-TNFRSF25 receptor-ligand pairs increased between malignant cells with high KIF18B expression and Tregs, suggesting that KIF18B activates Tregs by upregulating ICOSL and TNFSF15, which stimulate the co-stimulatory signal receptors CD28, ICOS, and TNFRSF25 (62–64). High KIF18B expression also enhanced the expression of the chemokines, CXCL10 and CXCL11, which attracted CXCR3^+^ Tregs to the TME (65). Moreover, upregulated LGALS9-CD44 signaling may improve Tregs stability and function (66). Recent studies have shown that HAVCR2, a modular receptor with multiple co-inhibitory receptors (e.g., checkpoint receptors), is present on exhausted T cells in various cancers (67). In addition, blocking HAVCR2 and PD-1 caused tumor regression in preclinical models and boosted anti-tumor T cell responses in patients with advanced cancer (68, 69). In this study, LGALS9-HAVCR2 expression in malignant cells with high KIF18B expression and Tregs was high, and this receptor-ligand pair has been shown to increase the immunosuppressive activity of Tregs (67). In summary, the stimulatory interaction and immune checkpoint modulation between malignant cells and CD4^+^ Tregs may account for activated Tregs infiltration in the high KIF18B expression group, promoting tumor growth and progression.

Interestingly, the expression of exhaustion genes, especially LAG-3 and HAVCR2, was significantly negatively correlated with KIF18B expression. LAG-3 is an inhibitory receptor that binds to major histocompatibility complex class II molecules and negatively regulates T-cell activation (70). Blocking LAG-3 enhances proliferation and effector functions of cytotoxic T lymphocytes. HAVCR2 is a critical surface protein in exhausted T cells that can dampen immune responses (67, 71). In NPC, LAG-3 and HAVCR2 are strongly associated with intra-tumoral TILs, and exhibit activation-dependent exhaustion expression patterns (33, 72, 73). This accounts for their inverse correlation with KIF18B and suggests that they may serve as promising therapeutic checkpoint targets in the KIF18B high-expression group.

The critical involvement of EMT in tumor development, invasion, and metastasis has been previously highlighted (74). In this study, GSEA demonstrated that EMT was a prominent characteristic of the high KIF18B expression group. Moreover, our observations revealed that KIF18B was associated with EMT markers, ITGA6, VIM, and transcription factors ZEB1/2 (75). Likewise, prior research showed that KIF18B facilitated the progression of breast (41) and liver cancers (76) via EMT activation. Intriguingly, the enrichment of EMT pathway was also observed in Tregs within KIF18B-high group (**Supplementary Figure S5B**). The TME encompasses stromal cells that release various cytokines and chemokines directly into stroma or via exosomes (77). Secreted factors produced by Tregs act in a paracrine manner on adjacent cancer cells, potentially triggering the EMT program within them. In turn, as stromal cellular components induce EMT in cancer cells, the resultant quasi-mesenchymal tumor cells adapt by altering the behavior and activity of diverse cell types aggregated within the stroma. The expression of EMT-induced TGF-β and TSP1 can promote the formation of Tregs (74). The intricate interplay between KIF18B, EMT and Tregs warrants further investigation.

M6A RNA methylation, the third layer of epigenetics, is the most predominant and abundant internal modification in eukaryotic cells (78). It alters RNA post-transcriptionally and influences its transport, splicing, degradation, and translation (78). Accumulating evidence suggests that m6A is frequently dysregulated in different cancers, augmenting tumorigenesis as well as tumor immune evasion (35, 79). In this study, we discovered that KIF18B expression was positively associated with most m6A “writer” and “reader” genes and negatively associated with “eraser” genes. Given the elevated levels of KIF18B mRNA and protein expression, as well as the correlation between KIF18B and m6A methylation, we hypothesized that m6A modification was a likely driver of KIF18B aberrant overexpression in NPC. Further analyses demonstrated the prognostic value of ELAVL1 in patients with NPC. ELAVL1 promotes the expression of certain tumor-related genes, such as ZMYM1 and DRG1, in an m6A-dependent manner, thereby exerting pro-carcinogenic effects (80, 81). Current research on KIF18B and its methylation mainly examines the methylation of KIF18B DNA. However, little is known about the manner in which m6A modification regulates KIF18B expression (82). Overall, we predict that ELAVL1 mediates m6A modification of KIF18B.

This study demonstrated that KIF18B was highly expressed in NPC and that such high KIF18B expression was correlated with unfavorable prognosis. Our findings suggested that the oncogenic impact of KIF18B in NPC was plausibly mediated by the activation of EMT and facilitation of immune evasion, and that the dysregulation of KIF18B may be modulated by m6A methylation. Moreover, we were able to unveil the potential mechanisms underlying KIF18B-mediated interactions between tumor cells and adjacent cellular components. Overall, the findings of this study indicate that KIF18B shows potential as a novel prognostic marker or therapeutic target in NPC.

## Data availability statement

The original contributions presented in the study are included in the article/**Supplementary Material**. Further inquiries can be directed to the corresponding authors.

## Ethics statement

The studies involving humans were approved by Ethics Committee of First People’s Hospital of Foshan. The studies were conducted in accordance with the local legislation and institutional requirements. The participants provided their written informed consent to participate in this study.

## Author contributions

ST: Conceptualization, Data curation, Formal Analysis, Funding acquisition, Investigation, Methodology, Resources, Software, Writing – original draft, Writing – review & editing. ZW: Conceptualization, Software, Writing – review and editing. LC: Data curation, Funding acquisition, Resources, Writing – original draft. LS: Data curation, Funding acquisition, Resources, Writing – original draft. WZ: Formal Analysis, Funding acquisition, Writing – original draft. WL: Formal Analysis, Funding acquisition, Writing – original draft. YZ: Investigation, Writing – original draft. SL: Methodology, Writing – original draft. GL: Methodology, Supervision, Writing – original draft. BH: Resources, Writing – original draft. JH: Resources, Writing – original draft. NZ: Supervision, Writing – review and editing.
