# Supplementary material for: Multi-omics analysis reveals the association between elevated KIF18B expression and unfavorable prognosis, immune evasion, and regulatory T cell activation in nasopharyngeal carcinoma
Source: Front Immunol. 2023 Sep 8;14:1258344. doi: 10.3389/fimmu.2023.1258344 (PMC10514916; doi:10.3389/fimmu.2023.1258344)
Supplement: Supplementary file 1 [file DataSheet_1.docx]

***Supplementary Material***

Multi-omics analysis reveals the association between elevated KIF18B expression and unfavorable prognosis, immune evasion, and regulatory T cell activation in nasopharyngeal carcinoma

**Siqi Tang*, Zhenyu Wu, Lusi Chen, Longjiang She, Weihan Zuo, Weijun Luo, Yang Zhang, Shaoqiang Liang, Guichao Liu, Biyi He, Jinfeng He, Ning Zhang^*^**

*** Correspondence:**Siqi Tang
tangsq3@mail2.sysu.edu.cn

Ning Zhang
zning@fsyyy.com

**1 Supplementary Figures**


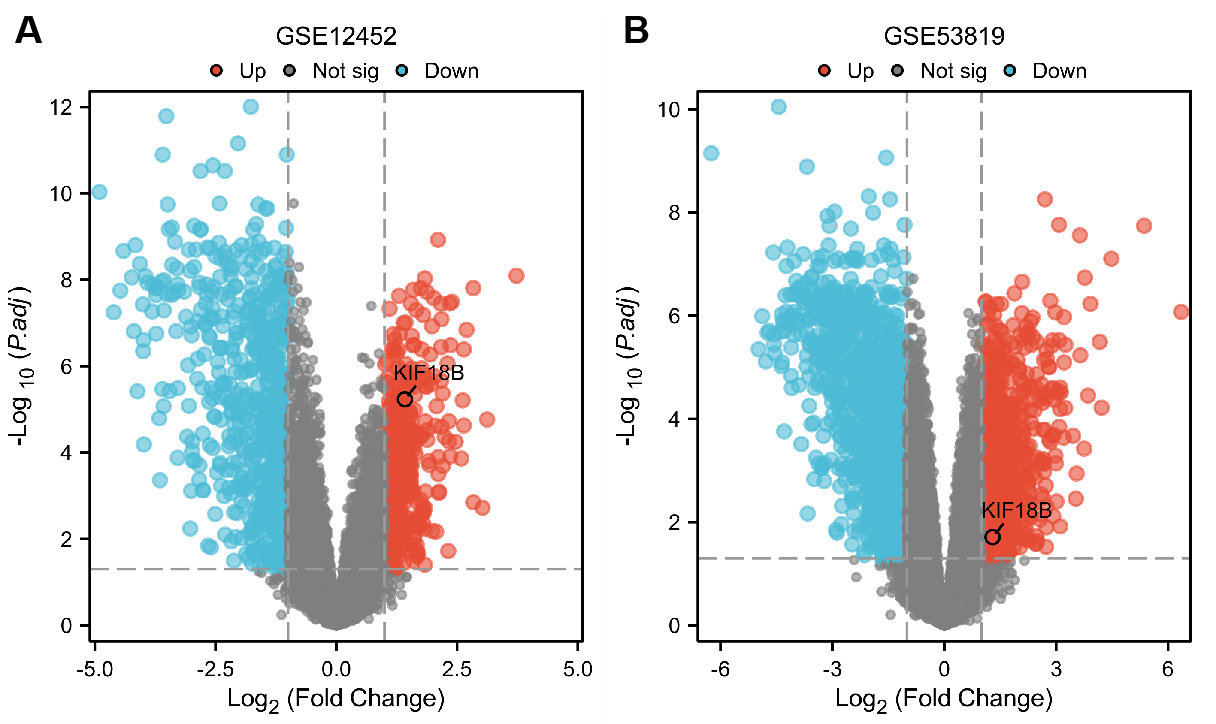


**Supplementary Figure S1.** Volcano map of differential expression genes between nasopharyngeal carcinoma and non-maligant nasopharyngeal tissues in **(A)** GSE12452 and **(B)** GSE53819 cohort. The horizontal dashed line represents adjust *P*-value equal to 0.05. The vertical dashed lines represent log_2_(Fold change) equal to -1 and 1, respectively.


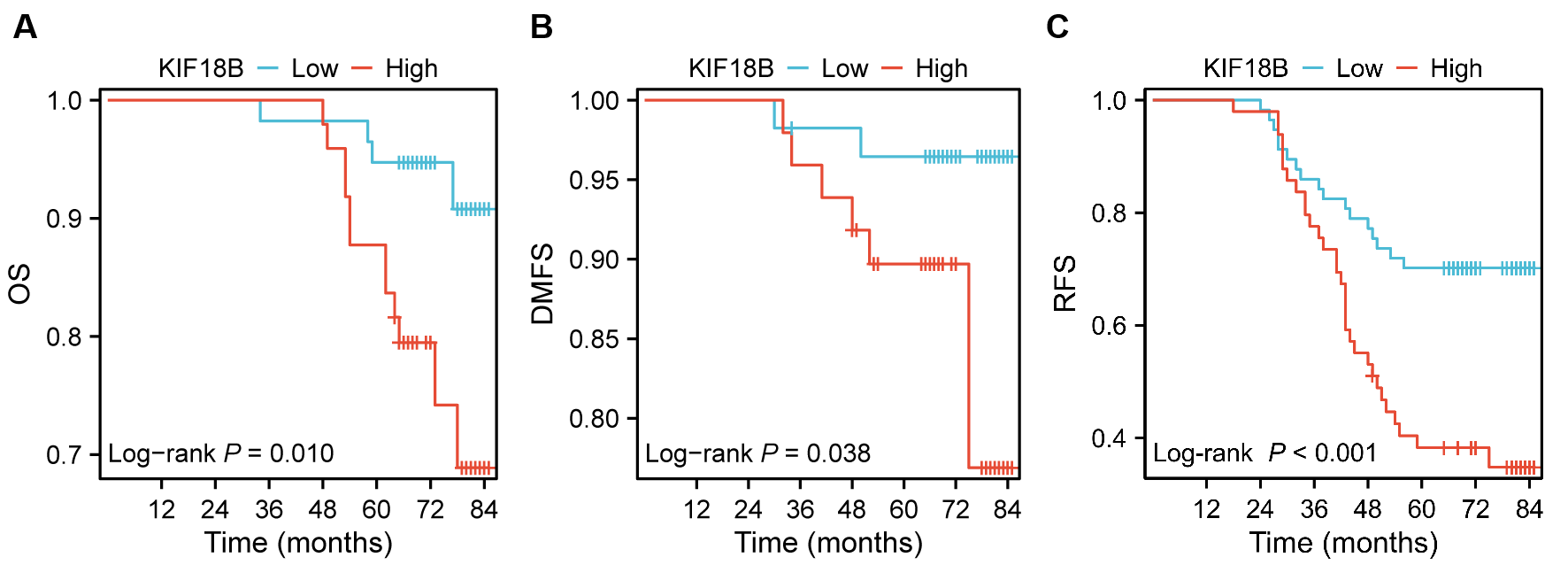


**Supplementary Figure S2.** The Kaplan-Meier survival analysis of OS **(A)**, DMFS **(B)**, and RFS **(C)** in patients with nasopharyngeal carcinoma (low *n* = 57, high *n* =49). OS, overall survival; DMFS, distant metastasis-free survival; RFS, recurrence-free survival.


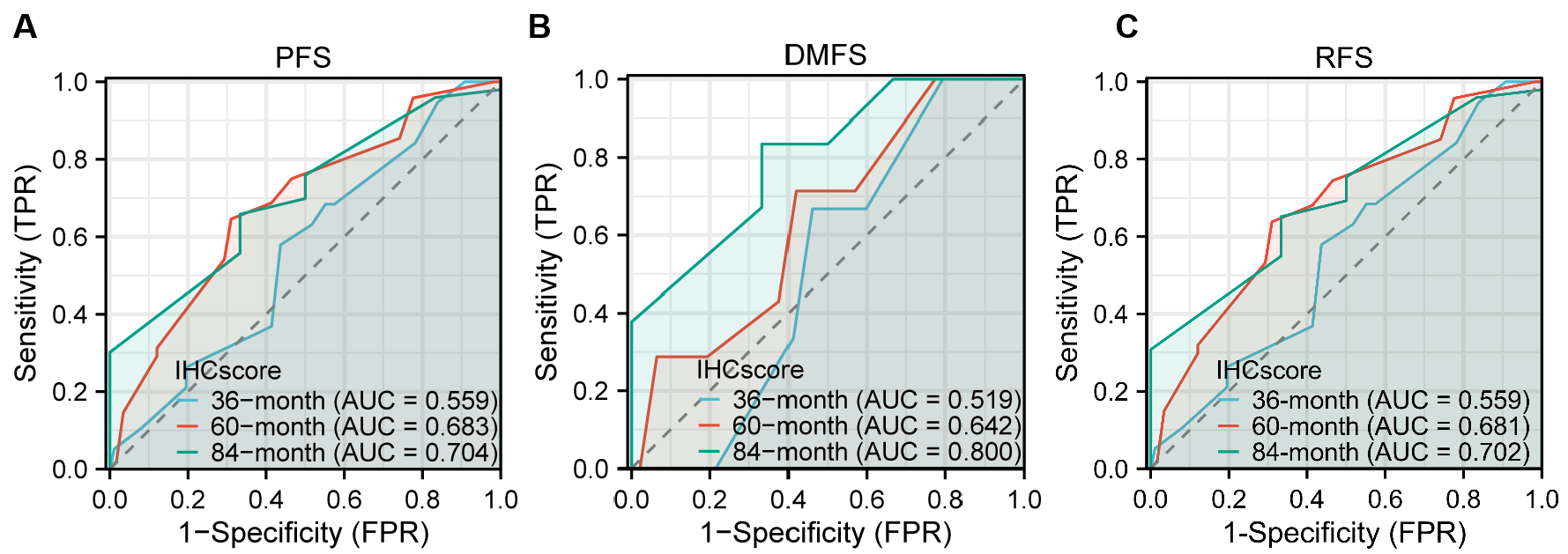


**Supplementary Figure S3.** The diagnostic power of KIF18B protein for PFS **(A)**, DMFS **(B)**, and RFS **(C)** outcomes. PFS, progression-free survival; DMFS, distant metastasis-free survival; RFS, recurrence-free survival; AUC, area under curve.


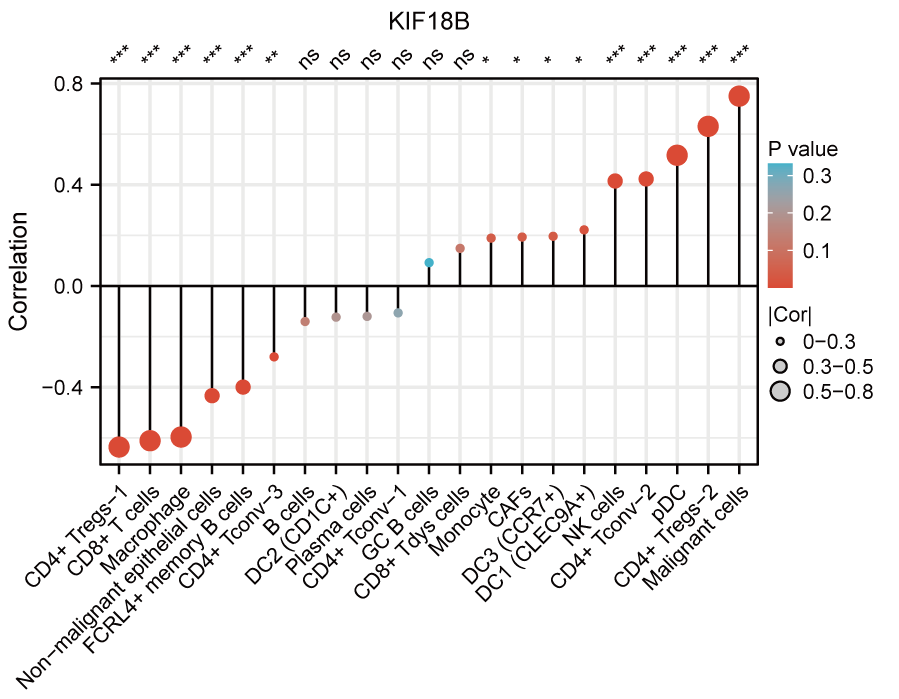


**Supplementary Figure S4.** Correlation analysis between KIF18B expression and infiltration level of different cell types. **P* < 0.05, ***P* < 0.01, ****P* < 0.001; ns, not significant; CD4+ Tregs, regulatory CD4^+^ T cells; CD4+ Tconv, conventional CD4^+^ T cells; CD8+ Tdys cells, dysfunctional CD8^+^ T cells; NK, natural killer; GC, germinal center; pDC, plasmacytoid dendritic cell; DC, dendritic cell; CAFs, cancer-associated fibroblasts.


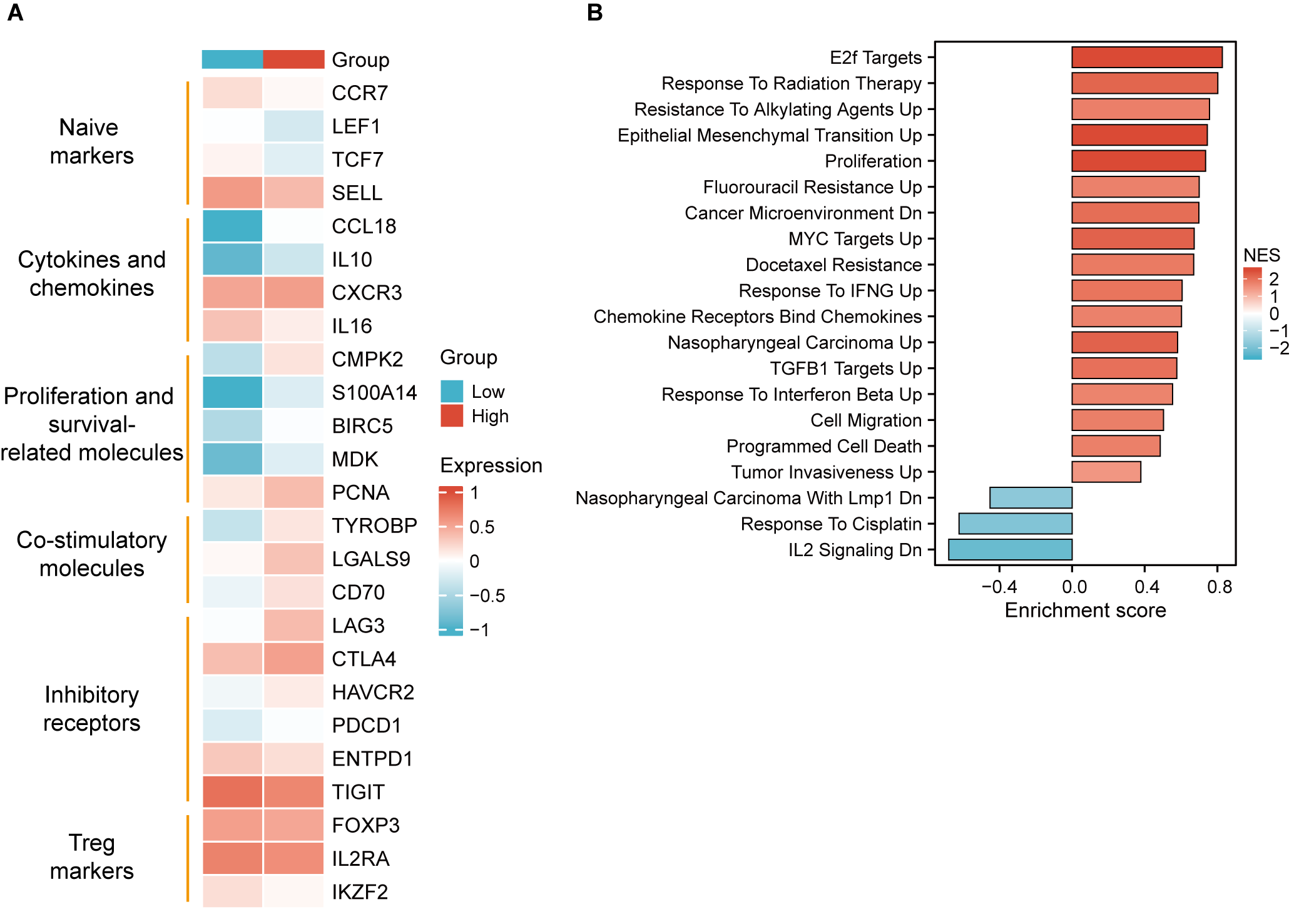


**Supplementary Figure S5. (A)** Average expression of selected T cell function-associated genes of naïve markers, cytokines and chemokines, proliferation and survival-related molecules, co-stimulatory molecules, inhibitory receptors, and Treg markers in each Treg subpopulations from KIF18B-low and -high groups. **(B)** Biological analysis on Treg subpopulations from KIF18B-high groups. Treg, regulatory T cell; NES, normalized enrichment score.

**
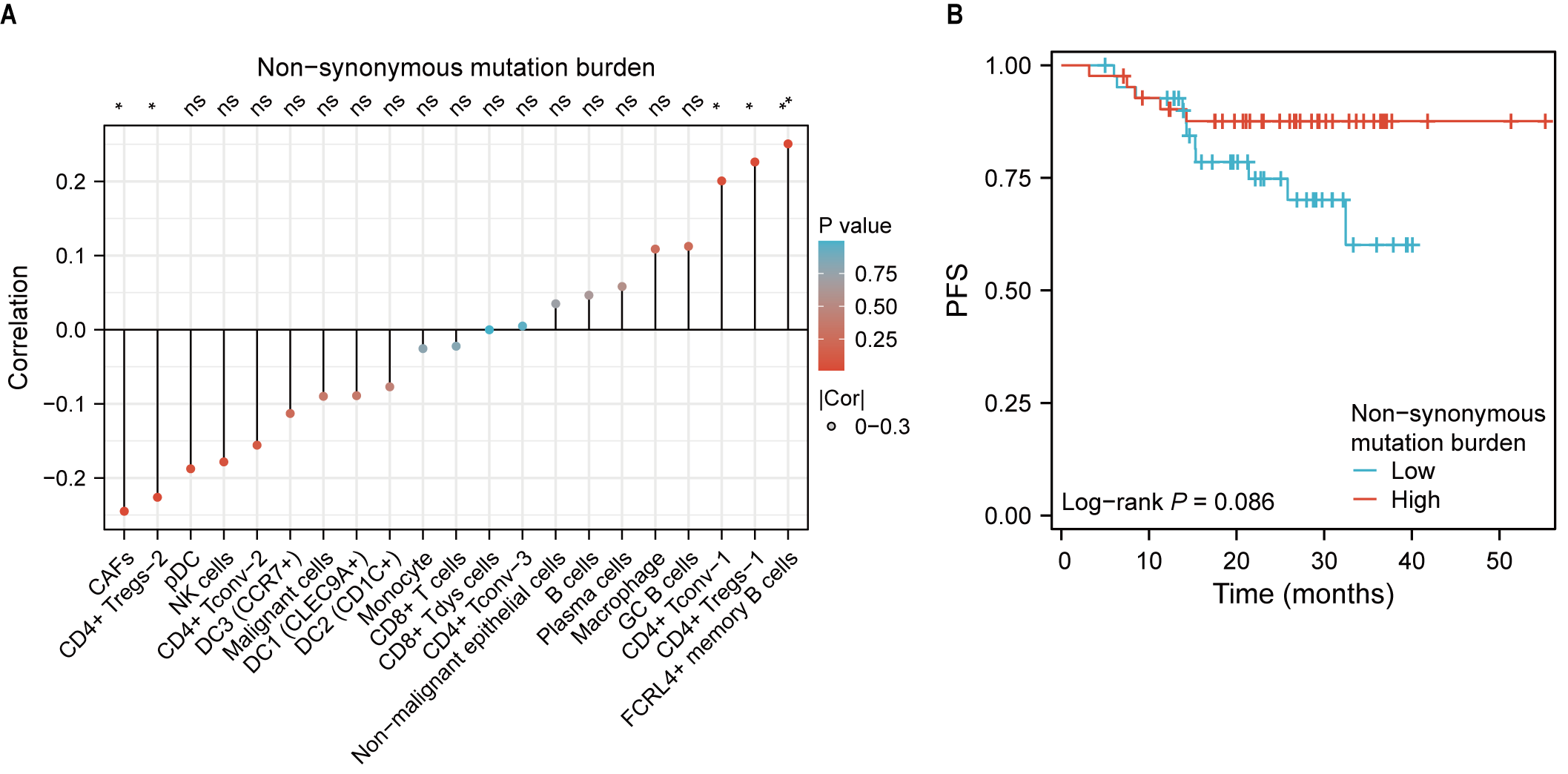
**

**Supplementary Figure S6.** **(A)** Correlation analysis between non-synonymous mutation burden and infiltration level of different cell types. **(B)** The Kaplan-Meier survival analysis of PFS in patients with different non-synonymous mutation burden (low *n* = 42, high *n* =42). **P* < 0.05, ***P* < 0.01; ns, not significant; CD4+ Tregs, regulatory CD4^+^ T cells; CD4+ Tconv, conventional CD4^+^ T cells; CD8+ Tdys cells, dysfunctional CD8^+^ T cells; NK, natural killer; GC, germinal center; pDC, plasmacytoid dendritic cell; DC, dendritic cell; CAFs, cancer-associated fibroblasts; PFS, progression-free survival.


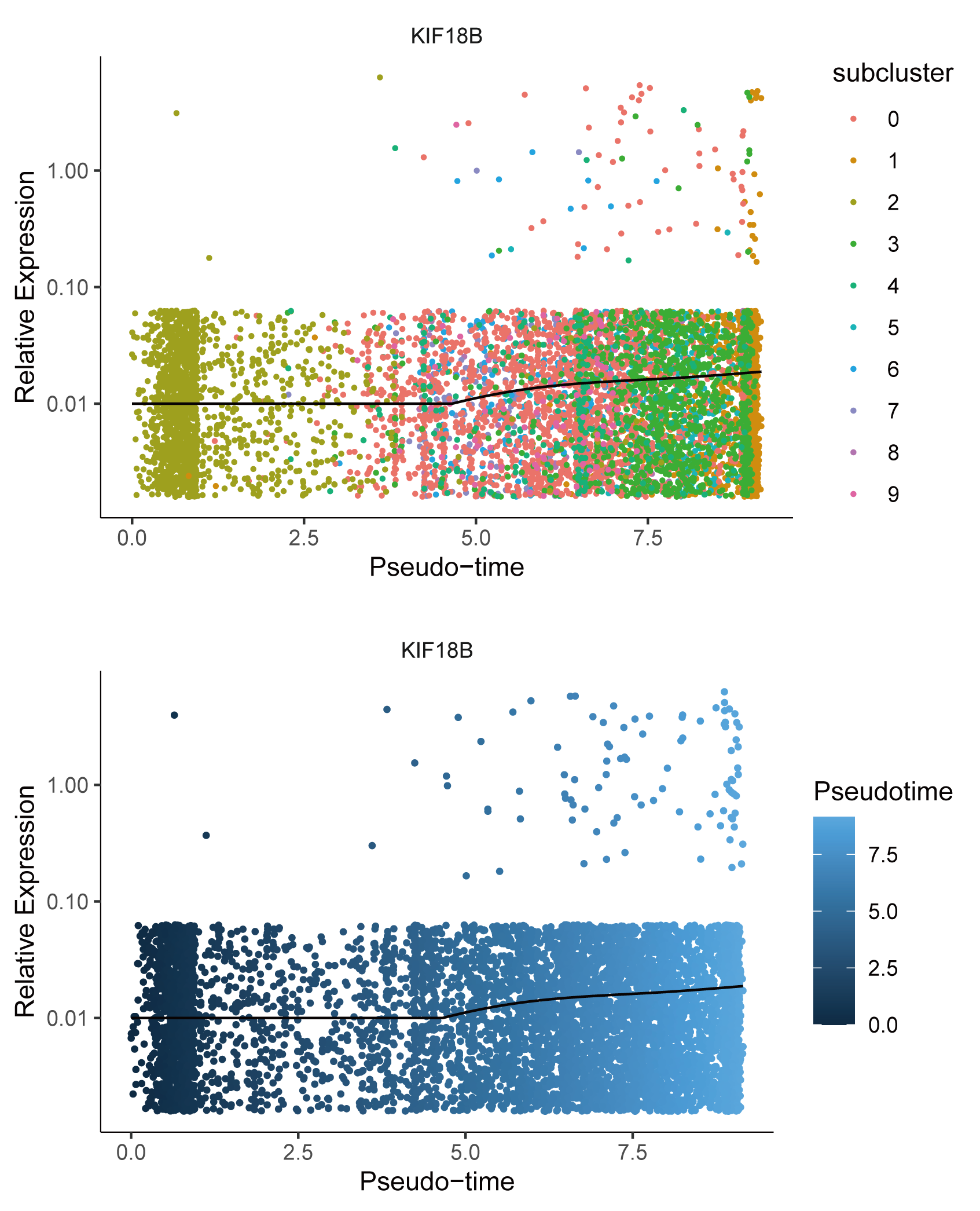


**Supplementary Figure S7.** Jitter plots showing the expression level of KIF18B changing with pseudotime.


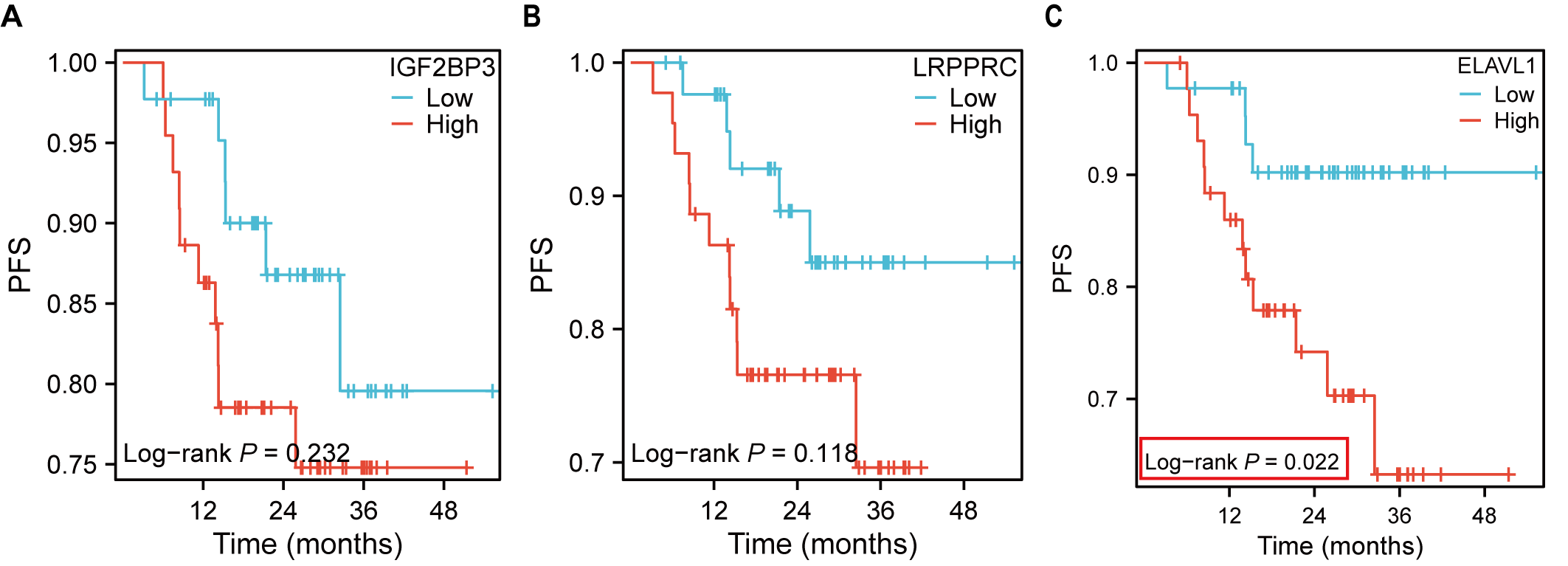


**Supplementary Figure S8.** The Kaplan-Meier survival analysis of PFS for nasopharyngeal carcinoma patients with different **(A)** IGF2BP3, **(B)** LRPPRC and **(C)** ELAVL1 expression in GSE102349 cohort (low *n* = 44, high *n* = 44). PFS, progression-free survival.


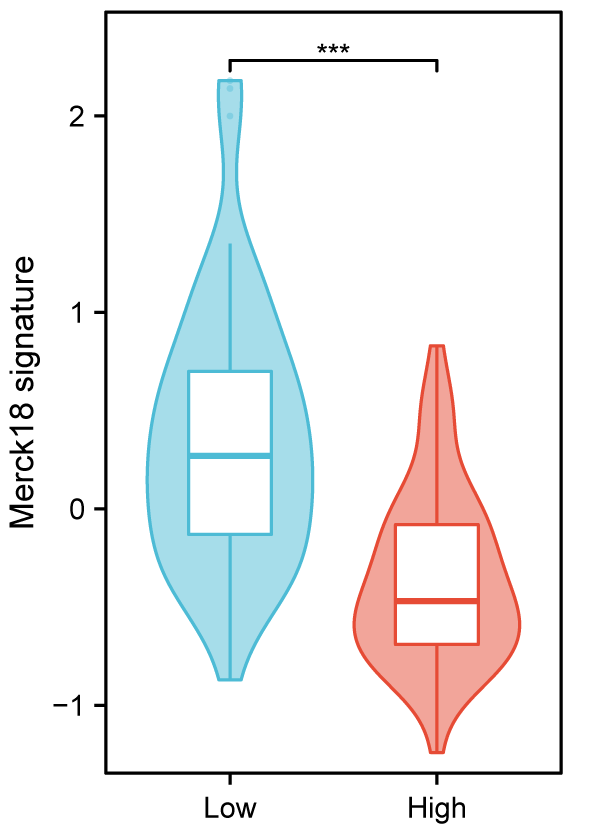


**Supplementary Figure S9.** Comparison of Merk18 signature score (Wilcoxon rank-sum test) between KIF18B-high and KIF18B-low group. ****P* < 0.001.

**2 Supplementary Tables S6-8**

**Supplementary Table S6.** Univariate and multivariate Cox hazard regression analysis of progression-free survival in GSE102349 dataset.

| Characteristics | Univariate analysis | |  | Multivariate analysis | |
| --- | --- | --- | --- | --- | --- |
|  | Hazard ratio (95% CI) | *P* value |  | Hazard ratio (95% CI) | *P* value |
| Stage |  | 0.160 |  |  |  |
| I-III | Reference |  |  |  |  |
| IV | 2.144 (0.749 - 6.136) | 0.155 |  |  |  |
| Stromal TILs |  | **0.010** |  |  |  |
| Low | Reference |  |  | Reference |  |
| High | 0.221 (0.061 - 0.798) | 0.021 |  | 0.172 (0.045 - 0.655) | **0.010** |
| Intratumoral TILs |  | 0.345 |  |  |  |
| Low | Reference |  |  |  |  |
| High | 0.557 (0.155 - 2.000) | 0.370 |  |  |  |
| Morphologic subtype |  | 0.751 |  |  |  |
| Undifferentiated, round | Reference |  |  |  |  |
| Undifferentiated, spindle | 0.989 (0.199 - 4.925) | 0.989 |  |  |  |
| Mixed (round & spindle) | 0.519 (0.146 - 1.844) | 0.311 |  |  |  |
| Differentiated | 0.711 (0.143 - 3.542) | 0.677 |  |  |  |
| Non-synonymous mutation burden |  | **0.084** |  |  |  |
| Low | Reference |  |  | Reference |  |
| High | 0.407 (0.141 - 1.176) | 0.097 |  | 0.197 (0.051 - 0.763) | **0.019** |
| KIF18B |  | **0.003** |  |  |  |
| Low | Reference |  |  | Reference |  |
| High | 5.218 (1.483 - 18.356) | 0.010 |  | 10.455 (2.249 - 48.604) | **0.003** |

Variates with a *P*-value ≤ 0.1 in the univariate analysis were highlighted in bold and included in the subsequent multivariate analysis. A high α level as the threshold of significance is acceptable in reducing the risk of discarding potentially validated factors. CI, confidence interval; TILs, tumor-infiltrating lymphocytes.

**Supplementary Table S7.** Prediction of N6-methyladenosine (m6A) modification site for KIF18B transcript variant 1

| No. | Position | Sequence context | Structural context | Score  (binary) | Score(knn) | Score(spectrum) | Score(combined) | Confidence |
| --- | --- | --- | --- | --- | --- | --- | --- | --- |
| 1 | 565 | UCAUGUACCUGACCACCGUGGAACUGUACAGGCGCCUGGAGGCCC | HHHHPPMPPPIIPPPIPPPPPIIIPPPIPIPPPMPPPPPIPPPPP | 0.645 | 0.679 | 0.422 | 0.557 | Low |
| 2 | 721 | ACAAGGGGGUGGUGGUGCAAGGACUUUCUUUCCACCAGCCAGCCU | MMMMPPPPIPPPPPPPPIPPPPHHHHHPPPPIPPPPPIPPPIPPP | 0.752 | 0.792 | 0.472 | 0.642 | High |
| 3 | 880 | AGCAGCAGGACCGGGUUCCAGGACUGACCCAGGCUGUCCAGGUGG | IPPPPPPPPIIPPPPPPMMMPPHHHHHHPPMMPPPPPPPIPPPPP | 0.738 | 0.777 | 0.402 | 0.606 | Moderate |
| 4 | 1074 | AAGACCCAUGUGCCCUACCGGGACAGCAAACUGACCCGCCUGCUC | MMMMMMMMPPIPPIIIIIPPPPIPPPHHHHPPPIPPPPMMMPPIP | 0.544 | 0.607 | 0.647 | 0.588 | Moderate |
| 5 | 1101 | AAACUGACCCGCCUGCUCAAAGACUCCCUCGGGGGCAACUGCCGC | HHHPPPIPPPPIIIPPIPPMMMMMMMPPPBPPPPPPMMMMPPIPP | 0.641 | 0.857 | 0.514 | 0.601 | Moderate |
| 6 | 1248 | AAGAGCAAUGUGACCAGCCUGGACUGUCACAUCAGCCAGUAUGCU | IIPBPPPPPPIIPIPPIIPPPPBPPPHHHHHHPPPPPPPIIPPIP | 0.758 | 0.711 | 0.427 | 0.623 | High |
| 7 | 1829 | GCCUGGGGCAGAGGCCUUGAGGACUUCAGGCCUGGCCAGGGGGGC | PPMPPPPPPPPMPPIPPPPBPPBPPIPPPPIIIPPPMPPPPPPMM | 0.605 | 0.497 | 0.599 | 0.597 | Moderate |
| 8 | 1943 | AUACACUGGCCCUGUGACCCGGACUAUGGCGAGGCGACUGAGUGG | PPPIIPPPPPMMMPPPPPPPPMMMMMMPPPPBPPPPPIIIIPPPP | 0.648 | 0.801 | 0.552 | 0.617 | Moderate |
| 9 | 2787 | CUCUGCUGGAUACCCCUCUUGGACCUGUAGCCACCUGCACCAGGA | HHHHPPPPPMMMMMMPPPPPPPBBBPPPPPHHHHPPPPPPPPPPP | 0.845 | 0.532 | 0.801 | 0.812 | Very high |

**Supplementary Table S8.** Prediction of N6-methyladenosine (m6A) modification site for KIF18B transcript variant 2

| No. | Position | Sequence context | Structural context | Score  (binary) | Score(knn) | Score(spectrum) | Score(combined) | Confidence |
| --- | --- | --- | --- | --- | --- | --- | --- | --- |
| 1 | 565 | UCAUGUACCUGACCACCGUGGAACUGUACAGGCGCCUGGAGGCCC | HHHHPPMPPPIIPPPIPPPPPIIIPPPIPIPPPMPPPPPIPPPPP | 0.645 | 0.679 | 0.422 | 0.557 | Low |
| 2 | 721 | ACAAGGGGGUGGUGGUGCAAGGACUUUCUUUCCACCAGCCAGCCU | IPPIPPMPPPPPPPPPMMMMPPPHHHHHHHPPPMPPPPPIPPPPP | 0.752 | 0.792 | 0.472 | 0.642 | High |
| 3 | 880 | AGCAGCAGGACCGGGUUCCAGGACUGACCCAGGCUGUCCAGGUGG | PPIIPPPPPPIPPPPBBBBPPPPPPPPIPPPPBBBBBPPPPPPPP | 0.738 | 0.777 | 0.402 | 0.606 | Moderate |
| 4 | 1074 | AAGACCCAUGUGCCCUACCGGGACAGCAAACUGACCCGCCUGCUC | MMMMMMMMPPIPPIIIIIPPPPIPPPHHHHPPPIPPPPMMMPPIP | 0.544 | 0.607 | 0.647 | 0.588 | Moderate |
| 5 | 1101 | AAACUGACCCGCCUGCUCAAAGACUCCCUCGGGGGCAACUGCCGC | HHHPPPIPPPPIIIPPIPPMMMMMMMPPPBPPPPPPMMMMPPIPP | 0.641 | 0.857 | 0.514 | 0.601 | Moderate |
| 6 | 1248 | AAGAGCAAUGUGACCAGCCUGGACUGUCACAUCAGCCAGUAUGCU | IIPBPPPPPPMMPIPPMMPPPPBPPPHHHHHHPPPPPPPMMPPIP | 0.758 | 0.711 | 0.427 | 0.623 | High |
| 7 | 1865 | GCCUGGGGCAGAGGCCUUGAGGACUUCAGGCCUGGCCAGGGGGGC | PPIPPPPPPPPMPPBPPPPBPPIPPIPPPPIIIPPPIPPPPPPMM | 0.605 | 0.497 | 0.599 | 0.597 | Moderate |
| 8 | 1979 | AUACACUGGCCCUGUGACCCGGACUAUGGCGAGGCGACUGAGUGG | IPIPPIPPPPPPPPPPIPPPPPIPPPMPPPPBPPPPPIIIIPPPP | 0.648 | 0.801 | 0.552 | 0.617 | Moderate |
| 9 | 2652 | GGGCCCCUUGUACUCCCAGGUGACUGGCACUAGGGACAGGGAUAG | PPPMMPPPPPPBPPPPPPPHHHHPPPPIIIIIPPPPPPPPPMPPP | 0.832 | 0.735 | 0.895 | 0.852 | Very high |
| 10 | 2665 | UCCCAGGUGACUGGCACUAGGGACAGGGAUAGCCUGGGCCAUGGA | PPPPPPHHHHPPPPIIIIIPPPPPPPPPMPPPIPPPPPIPPPPPB | 0.587 | 0.624 | 0.678 | 0.625 | High |
| 11 | 3050 | CUCUGCUGGAUACCCCUCUUGGACCUGUAGCCACCUGCACCAGGA | HHHHPPPPPMMMMMMPPPPPPPBBBPPPPPHHHHPPPPPPPPPPP | 0.845 | 0.532 | 0.801 | 0.812 | Very high |
